# Chronic nicotine exposure induces molecular and transcriptomic endophenotypes associated with mood and anxiety disorders in a cerebral organoid neurodevelopmental model

**DOI:** 10.3389/fphar.2024.1473213

**Published:** 2024-12-23

**Authors:** Emma K. Proud, Mar Rodríguez-Ruiz, Dana M. Gummerson, Sebastian Vanin, Daniel B. Hardy, Walter J. Rushlow, Steven R. Laviolette

**Affiliations:** ^1^ Addiction Research Group, Schulich School of Medicine and Dentistry, University of Western Ontario, London, ON, Canada; ^2^ Department of Anatomy and Cell Biology, University of Western Ontario, London, ON, Canada; ^3^ St. Jopsephs Health Care, Lawson Health Research Institute, London, ON, Canada; ^4^ Department of Physiology and Pharmacology, Western University, London, ON, Canada; ^5^ Department of Obstetrics and Gynecology, Western University, London, ON, Canada; ^6^ St. Josephs Healthcare, Children’s Health Research Institute, London, ON, Canada; ^7^ Department of Psychiatry, University of Western Ontario, London, ON, Canada

**Keywords:** prenatal development, prenatal nicotine exposure, anxiety, depression, human cerebral organoids

## Abstract

**Introduction:**

Prenatal nicotine exposure (PNE) from maternal smoking disrupts regulatory processes vital to fetal development. These changes result in long-term behavioral impairments, including mood and anxiety disorders, that manifest later in life. However, the relationship underlying PNE, and the underpinnings of mood and anxiety molecular and transcriptomic phenotypes remains elusive.

**Methods:**

To model nicotine exposure during prenatal development, our study used human cerebral organoids that were chronically exposed to nicotine and collected for molecular analyses.

**Results:**

Short-term, nicotine altered molecular markers of neural identity, mood and anxiety disorders and those involved in maintaining the excitatory/inhibitory (E/I) balance in the cortex. RNA sequencing further revealed transcriptomic changes in genes pertaining to embryonic development, neurogenesis, and DNA binding. Long-term, mature organoids demonstrated similar disruptions in E/I balance, decreased expression of neural identity markers, and altered dopamine receptor expression.

**Discussion:**

Collectively, our results demonstrate that nicotine-induced alterations occur acutely and persist at later stages of development. These findings validate an *in vitro* model of PNE to better comprehend the emergence of neuropsychiatric molecular and transcriptomic endophenotypes resulting from gestational nicotine exposure.

## Introduction

Rates of cigarette smoking have declined substantially in recent years. However, the use of electronic nicotine delivery systems such as vapes and e-cigarettes has been increasing exponentially, including in women of reproductive age (Brooks and Henderson, 2021; Obisesan et al., 2020). These trends are alarming given that nicotine has been reported to disrupt several regulatory processes vital to healthy fetal development (Dwyer et al., 2009). As a result, prenatal nicotine exposure (PNE) has been linked to numerous physical and emotional disturbances that persist later into the life of the offspring (Blood-Siegfried and Rende, 2010; Dwyer et al., 2009).

Nicotinic acetylcholine receptors (nAchRs) are critical in facilitating key aspects of prenatal neurodevelopment such as neurogenesis, cell survival, apoptosis, and axonal and synaptic growth (Ekblad et al., 2010; Smith et al., 2010). Cholinergic signaling plays a crucial role in coordinating brain maturation and premature or chronic overactivation of nAchRs during this critical period of neurodevelopment interferes with these regulatory processes (Dwyer et al., 2009; Moylan et al., 2013). This produces detrimental changes in nAchR distribution, sensitivity, and neurotransmitter functions, which can lay the foundation for future mood and anxiety disorders (Dwyer et al., 2009; Laviolette, 2021; Mahar et al., 2012; Sailer et al., 2019). Specifically, the most abundant nAchRs in the cortex implicated in mood and anxiety disorders are the ⍺_7_ and ⍺_4_β_2_ subunits. Nicotine also modulates the release and signaling of several neurotransmitter systems within the central nervous system, such as glutamate, γ-aminobutyric acid (GABA) and dopamine (Mahar et al., 2012; Moylan et al., 2013; Sailer et al., 2019). For example, ⍺_7_ nAchRs located presynaptically on glutamatergic neurons indirectly mediate the release of dopamine from neighboring neurons, which alters dopamine signaling and excitatory/inhibitory (E/I) balance in the cortex (Livingstone et al., 2010). Prenatal neurogenesis is a precise series of synchronized events during which the brain is tremendously vulnerable to external environmental stimuli, like nicotine exposure (Ross et al., 2015). This exposure produces perpetual alterations in neuronal cytoarchitecture and brain circuity of the fetus in brain regions vital for emotional regulation, such as the prefrontal cortex (PFC), with adverse neurobehavioral outcomes persisting into adulthood (Aoyama et al., 2016; Blood-Siegfried and Rende, 2010; Dwyer et al., 2019; Laviolette, 2021; Mahar et al., 2012; Minatoya et al., 2019; Moylan et al., 2015; Sailer et al., 2019; Smith et al., 2010).

Rodent models have provided considerable insights in identifying biomarkers associated with both mood and anxiety-like behaviors and developmental nicotine exposure (Hudson et al., 2021; Jobson et al., 2019; Polli et al., 2020; Slawecki et al., 2003; Smith et al., 2006; Vaglenova et al., 2004). Alterations in these biomarkers include changes in the expression of ⍺_7_ and ⍺_7_β_2_ nAchRs, dopamine 1 (D1R) and dopamine 2 (D2R) receptors (Hudson et al., 2021; Jobson et al., 2019; Mineur et al., 2011; Philip et al., 2010). Additional studies have also identified aberrant GABAergic and glutamate signaling as underlying factors in major-depressive disorder (MDD) and anxiety-related psychopathology. For example, cortical levels of glutamic acid decarboxylase (GAD67), GABA transporter type-1 (GAT-1), parvalbumin (PV) interneurons, N-methyl-D-aspartate and metabotropic (mGLUR) receptors are reportedly altered in *postmortem* and human imaging studies of patients with these disorders (Duman et al., 2019; Feyissa et al., 2010; Hashimoto, 2009; Hasler et al., 2007; Karolewicz et al., 2010; Rajkowska et al., 2007). Similarly, numerous differentially expressed genes (DEGs) involving GABAergic and glutamatergic neurotransmission have been reported in transcriptomic studies of patients with MDD, which further reinforces the association between altered E/I balance in the cortex and mood and anxiety disorders (Choudary et al., 2005; Klempan et al., 2009; Mehta et al., 2010; Sequeira et al., 2007; Sequeira et al., 2009). Furthermore, there is evidence of upregulation of genes encoding for proteins that facilitate transcription and translation in mood disorders, which is useful for understanding the genetic basis of molecular biomarkers and the pathophysiology of anxiety and depression (Iwamoto et al., 2004; Mehta et al., 2010).

Due to the paucity of access to fetal brain tissue, most of our knowledge regarding PNE, and the emergence of mood and anxiety disorders comes from clinical populations and animal models (Blood-Siegfried and Rende, 2010; Corrêa et al., 2022; Dwyer et al., 2009; Ekblad et al., 2010; Mahar et al., 2012; Minatoya et al., 2019; Moylan et al., 2013; Sailer et al., 2019; Smith et al., 2010). Although valuable, these models have many limitations due to confounding variables and species-specific differences in brain development, respectively. To bridge the gap between animal and two-dimensional *in vitro* models, three-dimensional cerebral organoids are proposed as an effective preclinical platform to recapitulate aspects of the developing human brain, in this case, in conjunction with nicotine exposure (Centeno et al., 2018; Kim et al., 2020; Lancaster et al., 2013). Previous organoid studies investigating the effect of nicotine exposure on prenatal brain development exist, but have not taken the emergence of mood and anxiety molecular endophenotypes into consideration (Notaras et al., 2021; Wang et al., 2018).

The present study aimed to validate an *in vitro* cerebral organoid model of PNE, to characterize how nicotine exposure early in pregnancy can lead to aberrant neurodevelopmental events and the emergence of molecular biomarkers of mood and anxiety disorders. We employed the use of immunofluorescence (IF), quantitative polymerase chain reaction (qPCR) and RNA sequencing (RNA-Seq) to identify different categories of biomolecules underlying how nicotine exposure contributes to molecular endophenotypes observed in mood and anxiety disorders’ pathologies. We report that nicotine altered established molecular markers pertaining to neural identity, mood and anxiety disorders and those involved in maintaining E/I balance in the cortex, with some of these effects persisting into later stages of development. Changes at the transcriptomic level were also reported. Observing these alterations in tandem validate our PNE model and further illuminate how the molecular mechanisms underlying nicotine exposure can alter human cortical brain development and dysregulate molecular pathways associated with mood and anxiety disorders.

## Materials and methods

### iPSC maintenance and organoid generation

Three human, control, induced pluripotent stem cell (iPSC) lines were acquired from deposits made to the National Institute of Mental Health Repository and Genomics Resource center and obtained from RUCDR Infinite Biologics. Each cell line (MH0185865, MH0185983 and MH0185984) originated from healthy patients without any history of neuropsychiatric disorders. The three iPSC cell lines included two males, aged 29, and one female, aged 24. They were chosen due to their age proximity and shared Caucasian background. The iPSC lines were maintained in hypoxic conditions (4% oxygen) until organoid generation and cultured concurrently to minimize variations from individual culturing practices. Briefly, for approximately 2 weeks, iPSCs were maintained in mTeSR™1 medium (StemCell, 85850) in Matrigel^®^ (Corning, 354277) coated plates and cells were passaged using Gentle Disassociation Reagent (StemCell, 07174) if colonies were roughly 70% confluent. To improve the success of cortical differentiation, for 4 days before starting the organoid protocol, the iPSCs were pretreated with mTeSR™1 containing different growth factors as previously described by Watanabe et al. (2019): bone morphogenetic protein 4 [(final) = 0.1 ng/mL], transforming growth factor beta-1 [(final) = 0.1 ng/mL], transforming growth factor beta-3 [(final) = 1 ng/mL] and activin-A [(final) = 10 ng/mL]. The intended starting confluency of the cell lines was approximately 70%.

Cerebral organoids were generated using the STEMdiff™ Cerebral Organoid Kit (StemCell, 08570) and the protocol was derived from Lancaster et al. (2013). On day 0 of organoid culture [embryoid body (EB) formation], iPSCs were lifted using Gentle Disassociation Reagent and gently triturated to create a single-cell suspension. Following centrifugation and resuspension, 100 µL of cell suspension (9,000 cells/well) was plated in each well of a 96-well round-bottom ultra-low attachment plate (Corning, 7007) with EB Formation Medium containing 10 µM rho-kinase inhibitor (StemCell, 72302). On days 2 and 4, 100 µL of EB Formation Medium was added to each well and the plate was incubated at 37°C until organoid induction on day 5.

On day 5, EBs were transferred to a 24-well ultra-low attachment plate (Corning, 3473), containing Induction Medium, that was pretreated with AggreWell™ Rinsing Solution (StemCell, 07010). The plates were incubated at 37°C for 48 h until organoid expansion on day 7.

On day 7, organoids were transferred to an Organoid Embedding Sheet (StemCell, 08579) and cold Matrigel was added dropwise onto each EB. To polymerize the Matrigel, the plate was placed in the incubator at 37°C for 30 min. Upon removing the plate from the incubator, EBs were washed from the Embedding Surface into 6-well plates containing Expansion Medium and the plates were incubated at 37°C for 3 days until organoid maturation on day 10.

**FIGURE 1:**
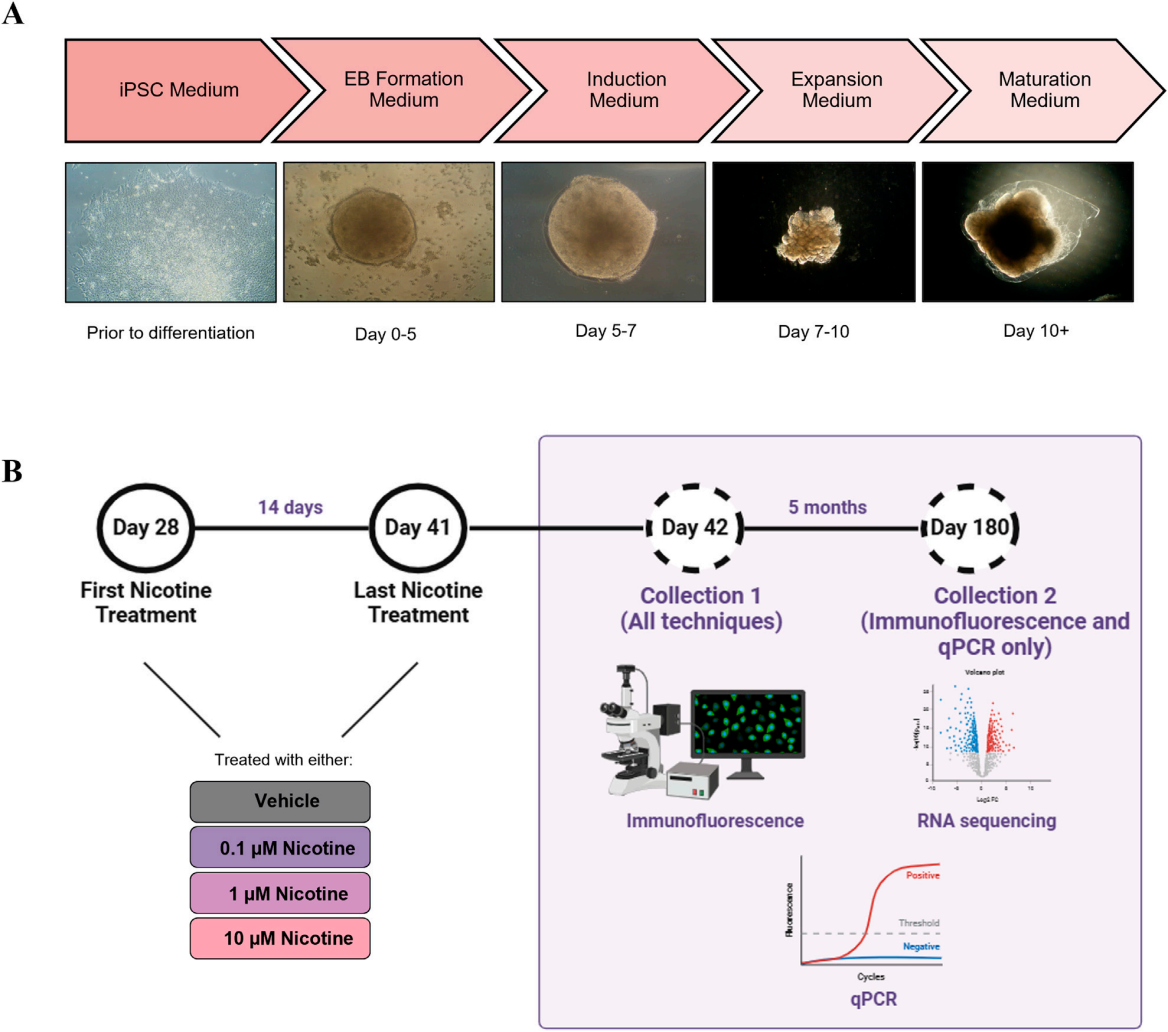
**(A)** Description of the cerebral organoid culture system and representative microscopic images. The iPSCs were maintained in culture for 2 weeks prior to organoid generation. On day 0, the iPSCs were cultured in an EB Formation Medium, and aggregated to form EBs. On day 5, the EBs were cultured in an Induction Medium which induced them to a neural fate. On day 7, the organoids were embedded in Matrigel and cultured in an Expansion Medium to allow expansion of the neuroepithelium. On day 10, the organoids were cultured in Maturation Medium and placed on an orbital shaker. Aside from nicotine treatment, when the medium was changed daily, the Maturation Medium was changed once every 3 days. **(B)** Details of nicotine treatment and organoid collection. Beginning on D28, the organoids were treated daily with VEH medium or medium containing one of three doses of nicotine, for 14 days. Following nicotine exposure, on D42 and at D180, the organoids were collected for IF, RNA-Seq and qPCR. Figure made using BioRender.

On day 10, all Expansion Medium was removed and replaced with 3 mL/well of Maturation Medium. The plates of organoids were placed on an orbital shaker and incubated in normoxic conditions at 37°C. Following day 10, Maturation Medium was changed every 3 days, except for days organoids received nicotine treatment (Figure 1). Beginning on day 48 (until approximately 4 months, 1 mL of Cultrex Reduced Growth Factor Basement Membrane Extract, Type 2 (R&D Systems, 3536-005-02) was added to each bottle of Maturation Medium to support organoid development and maturation. Following the four-month mark, the organoids received regular Maturation Medium until their final collection day.

### Nicotine treatment

Beginning on day 28, organoids were cultured in Maturation Medium with no nicotine [vehicle (VEH)], or a Maturation Medium treated with 0.1, 1 or 10 µM of nicotine for 14 days. The doses and the timing of nicotine treatment were based on studies conducted by Wang et al. (2018) and Notaras et al. (2021) to mimic physiological relevancy and organoid viability. The selected doses fell within the range of average serum concentrations previously reported in pharmacokinetic studies of cigarette smoking and/or nicotine replacement therapies (Deveaugh-Geiss et al., 2010; Massadeh et al., 2009; Oncken et al., 1997; Russell et al., 1980). Additional considerations influencing the selected developmental window of exposure included the active period of neurogenesis, timing of nAchR expression and recapitulating human brain development at early/mid gestation (Lancaster et al., 2013; Wang et al., 2018; Daviaud et al., 2019). To prepare the nicotine, a 100 µmol stock was created by dissolving nicotine hydrogen tartrate (Sigma, N5260) in sterile distilled water and 1 mL aliquots were kept at −20°C. On each day of nicotine treatment, an aliquot was thawed and diluted in Maturation Medium to reach the desired treatment concentrations. The Maturation Medium from all wells was aspirated, VEH or nicotine-treated medium was added to each respective well and the plate was incubated at 37°C for 24 h until the next drug treatment. Medium changes occurred daily for 14 days (the last treatment was on day 41). The organoids were collected for various histological and molecular techniques at day 42 (D42) and remained in culture until their final collection at day 180 (D180; Figure 1). Due to the prospective separation into the wells prior to nicotine treatment and the organoids being grown under the same experimental conditions, no specific criteria were used to select the organoids for each treatment. However, organoids that appeared similar in shape and size between conditions were selected for each experiment.

### Histology and immunofluorescence

The organoids were incubated in 4% paraformaldehyde, cryopreserved in 30% sucrose, embedded in 7.5% gelatin (Sigma, G2500) in embedding molds and cryosectioned at 20 µm. Tissue sections were stained with hematoxylin & eosin or used for IF. Hematoxylin & eosin staining was performed to confirm successful morphological development and neuronal induction (data not shown). Following sample preparation, slides were selected for staining and send to Pathology at Robarts Research Institute (Western University, Ontario, Canada). The slides were imaged using a brightfield microscope (Nikon H600L). Images were acquired with a 10x magnification and at 2880 × 2048 resolution.

Slides selected for IF were washed three times for 10 min in phosphate-buffered saline with 0.1% Tween^®^20 (PBS-T; Sigma, P9416) to completely remove the gelatin surrounding the organoids and an ImmEdge™ hydrophobic PAP pen (Vector, H-4000) was used to circle each organoid on the slide. The organoids were blocked in 5% normal donkey serum (Millipore, S30) in PBS-T in a humidified chamber for 1 h at room temperature. Primary antibodies were prepared in 5% donkey serum in PBS-T according to the recommended dilutions. Primary antibodies included ⍺_7_ nAchR (rabbit, Alomone labs ANC-007, 1:50), ⍺_4_ nAchR (mouse, Santa Cruz sc-74519, 1:50), β_2_ nAchR (goat, Abcam ab189174, 1:100), D1R (rabbit, Abcam ab40653, 1:50), D2R (mouse, Sigma-Aldrich MABN53, 1:200), mGLUR2/3 (rabbit, Sigma-Aldrich 06–676, 1:50), GAD67 (mouse, Sigma-Aldrich mab5406, 1:50), Ki67 (rabbit, Abcam ab15581, 1:500), PROX1 (mouse, Sigma-Aldrich mab5654, 1:100), FZD9 (goat, Abcam ab110886, 1:100), FGFR1 (rabbit, Abcam ab0646, 1:200), CCasp3 (rabbit, Cell signaling 9661, 1:200), CTIP2 (rat, abcam ab18465, 1:100), CDH13 (goat, Novus Biologicals, AF3264, 1:200), MAP2 (mouse, Sigma-Aldrich mab3418, 1:100), NR2B (goat, Novus Biologicals NB100-41097, 1:50), GAT-1 (rabbit, Rockland Immunochemicals 612-401-D56, 1:100) and PV (mouse, Sigma-Aldrich P3088, 1:50). For negative controls, the organoids remained covered with the blocking solution and received no primary antibody. The slides were incubated overnight at 4°C in humidified chambers. The slides were washed three times for 10 min in PBS-T, secondary antibodies were prepared in 5% donkey serum in PBS-T and sections were incubated at room temperature for 2 h in a humidified chamber covered from light. Secondary antibodies included rabbit (Alexa Fluor 488, A32790), mouse (Alexa Fluor 568, A10037), goat (Alexa Fluor 647, A21447) and rat (Alexa Fluor 647, A78947; Alexa Fluor 488, A21208). All secondary antibodies were used at a 1:250 dilution and obtained from Invitrogen. The slides were washed three times for 10 min in PBS-T, dried, mounted with FluoroShield™ (Sigma-Aldrich, F6057) containing DAPI and covered with a 1.5 mm coverslips (Fisherbrand, 12542A). The slides were stored at 4°C until imaging.

The organoids were imaged using a Leica SP8 (D42) or Leica STELLARIS5 (D180) microscope. The 40x and 63x magnifications were used on the Leica SP8 and STELLARIS5, respectively. Each treatment (i.e., VEH, and each nicotine dose) had one organoid collected for cryosectioning, per cell line. One section of organoid was selected for each experimental condition, for each combination of labels, and approximately two regions of interest (ROIs) were imaged per organoid. This was performed for each experimental condition, for each combination of labels. Images were taken in a 1024 × 1024 format, at a speed of 400 Hz. For each ROI, multiple optical z-slices were imaged and combined per microscopy image. Six steps ranging from 1.0–1.5 μm each, were taken per image of each ROI used for analysis. This method of imaging resulted in a range of 16–36 ROIs total (i.e., not per organoid) included for quantification, for each combination of labels. Data analysis for each marker of interest was performed using FIJI ImageJ (NIH). Regions of interest were manually selected, and images were normalized to the area by dividing the number of particles by the area (particles by mm^3^). Each marker of interest was analyzed at both time points, with nonsignificant data not shown.

### qPCR

Total RNA was extracted from whole organoids using the TRIizol (Invitrogen) and chloroform as specified by the manufacturer. Isopropanol was used to precipitate RNA, which was centrifuged to obtain a pellet. The pellet was dissolved in Diethyl pyrocarbonate (DEPC)-treated water. RNA was diluted to 1 μg/μL for reverse-transcriptase with a High-Capacity cDNA RT Kit (Applied Biosystems, 4368814) to make cDNA. The cDNA was diluted to 1:40 in qPCR.

Forward and reverse primers were designed using NCBI Primer Blast, and Harvard PrimerBank and sequences were validated using NIH Nucleotide Blast (Table 1). All primers were ordered from ThermoFisher, and quality was checked by analyzing the melt curves. DEPC blanks and 3 μL of cDNA were loaded into a 384 well plate (VWR, 82006-678) in triplicates. A master mix comprised of DEPC, 2.5 μM forward and reverse primer mix and SensiFAST SYBR (Meridian Bioscience, Bio-98050) was added to each well for a total reaction volume of 8 μL. Bio-Rad CFX384 Real-Time System was used with cyclic conditions set at 95°C for 3 min, followed by 43 cycles of; 95°C for 15 s, 58°C for 30 s and 72°C for 30 s. When the plate was finished running, the quality of the run was checked by examining the cycle quantification (Cq) values and melt curves. Values with a difference of > 0.5 Cq within a triplicate were removed and a Cq average was calculated for each sample. The values obtained for all gene targets of interest were normalized to the geometric means of housekeeping genes *ACTB* and *GAPDH*. *ACTB* and *GAPDH* were determined to be suitable housekeeping genes by using the comparative ΔCq method. The 2-ΔΔCq method was used to calculate the relative fold change of gene expression within the experimental samples. To enhance data transparency, ΔCt values for each primer were calibrated to experimental samples with the lowest transcript abundance (highest Ct value). Relative transcript abundance was then calculated for each primer set as determined by the formula 2^−ΔΔCT^, where ΔΔCt was the normalized value. Each target of interest was analyzed at both time points, with nonsignificant data not shown.

**TABLE 1:** qPCR primer sequences.

Gene	Forward sequence (5′-3′)	Reverse sequence (5′-3′)	Gene accession #
*D1R*	gac​ctt​gtc​tgt​act​cat​ctc​ct	gtc​aca​gtt​gtc​tat​ggt​ctc​ag	NM_000794.5
*EMX1*	cgc​agg​tga​agg​tgt​ggt​t	tcc​agc​ttc​tgc​cgt​ttg​t	NM_004097.3
*EOMES*	gtg​ccc​acg​tct​acc​tgt​g	cct​gcc​ctg​ttt​cgt​aat​gat	NM_001278182.2
*FOXG1*	agg​agg​gcg​aga​aga​aga​ac	tga​act​cgt​aga​tgc​cgt​tg	NM_005249.5
*GAD1*	gcg​gac​ccc​aat​acc​act​aac	cac​aag​gcg​act​ctt​ctc​ttc	NM_000817.3
*GRM2*	ccg​cat​tgc​acg​cat​ctt​c	ggc​ccg​aga​taa​gtg​cca​g	NM_000839.5
*ISL1*	gcg​gag​tgt​aat​cag​tat​ttg​ga	gca​ttt​gat​ccc​gta​caa​cct	NM_002202.3
*TBR1*	gac​tca​gtt​cat​cgc​cgt​ca	tgc​tag​tac​cct​agc​ctt​gc	NM_006593.4

### RNA-sequencing

Pooled male and female VEH (n = 3) and 0.1 µM nicotine-treated (n = 3) organoids were snap frozen and sent to Genome Quebec (Montreal, Quebec, Canada) for total RNA extraction, library preparation and RNA-Seq. Quality checks were performed by Genome Quebec following extraction and library preparation. The RNA integrity number was used to assess RNA quality and all samples had RNA integrity number scores ≥ 7.0. Paired-end reads (25 million) were sequenced on the Illumina NovaSeq platform. All raw reads were aligned and annotated with the latest ENSEMBL Homo Sapien GRCH38.p13 reference genome using STAR version 2.7.10a with recommended settings. Raw counts were generated using the Rsubread subpackage featureCounts (Liao et al., 2019). Lowly expressed genes were filtered out using a count per million (CPM) cutoff of 0.4 in at least two or more samples. Normalization and differential expression analysis were done using the edgeR package (Chen et al., 2016). Briefly, counts were normalized for both library size and library composition using the trimmed means of the M-values method. Normalized counts were then fit to a gene-wise negative binomial generalized linear model, and a quasi-likelihood F test was used for DE analysis. To account for multiple testing, *p*-values were adjusted using Benjamini & Hochberg False Discovery Rate (FDR) correction. An FDR cut-off of < 0.05 was used to determine significance. The gprofiler2 (Kolberg et al., 2020) R interface for the web toolset g: Profiler was used to convert ENSEMBL gene IDs to gene symbols, and to perform functional enrichment analysis (i.e., over-representation analysis) on the DEGs from databases of interest. The databases included in the analysis were the Gene Ontology (GO) database, the Reactome database, the TRANSFAC database, the human protein atlas and the WikiPathways database. A g: SCS adjusted *p*-value threshold of < 0.01 was used to determine the significance of the functional enrichment analysis. To examine DEGs between VEH and 0.1 µM nicotine organoids, a heatmap was generated using the pheatmat R package (Hu, 2021). Further analysis of DEGs was completed using VarElect (http://ve.genecards.org). VarElect is an application that permits the analysis of specific DEGs following sequencing and ranks genes that are found to have variants according to specific phenotype-gene associations. To generate this list, VarElect uses information obtained from several databases such as GeneCards^®^ (www.genecards.org), Malacards (www.malacards.org), LifeMap Discovery^®^ (discovery.lifemapsc.com) and Pathcards (pathcards.genecards.org). The list of DEGs was imported into VarElect and three phenotypes of interest were individually searched: nicotine exposure, anxiety, and depression. An annotated list of DEGs associated with each phenotype was generated. This list is formed based on direct (GeneCards) and indirect links (Genecards and Malacards) between the genes and phenotype of interest. The top 20 genes for each phenotype were compared to assess which DEGs were associated across multiple phenotypes.

### Statistical analyses

Outliers were removed using Grubbs’ test (α = 0.05) and normality was assessed. All results for IF and qPCR were analyzed with one-way analysis of variance (ANOVA) or Kruskal–Wallis if appropriate. Significant (*p* < 0.05) or trending (*p* < 0.1) tests were followed up using Fisher’s Least Squares Difference *post hoc* test (α = 0.05). All analyses were performed using GraphPad Prism (version 9.4.1 for Windows) and graphs are presented as mean ± standard error of the mean. For RNA-Seq, to account for multiple testing, *p*-values were adjusted using Benjamini & Hochberg FDR correction. An FDR cut-off of < 0.05 was used to determine significance.

## Results

### Short-term effects of PNE

#### Human cerebral organoids demonstrate successful neural induction and features of the developing fetal brain

**FIGURE 2:**
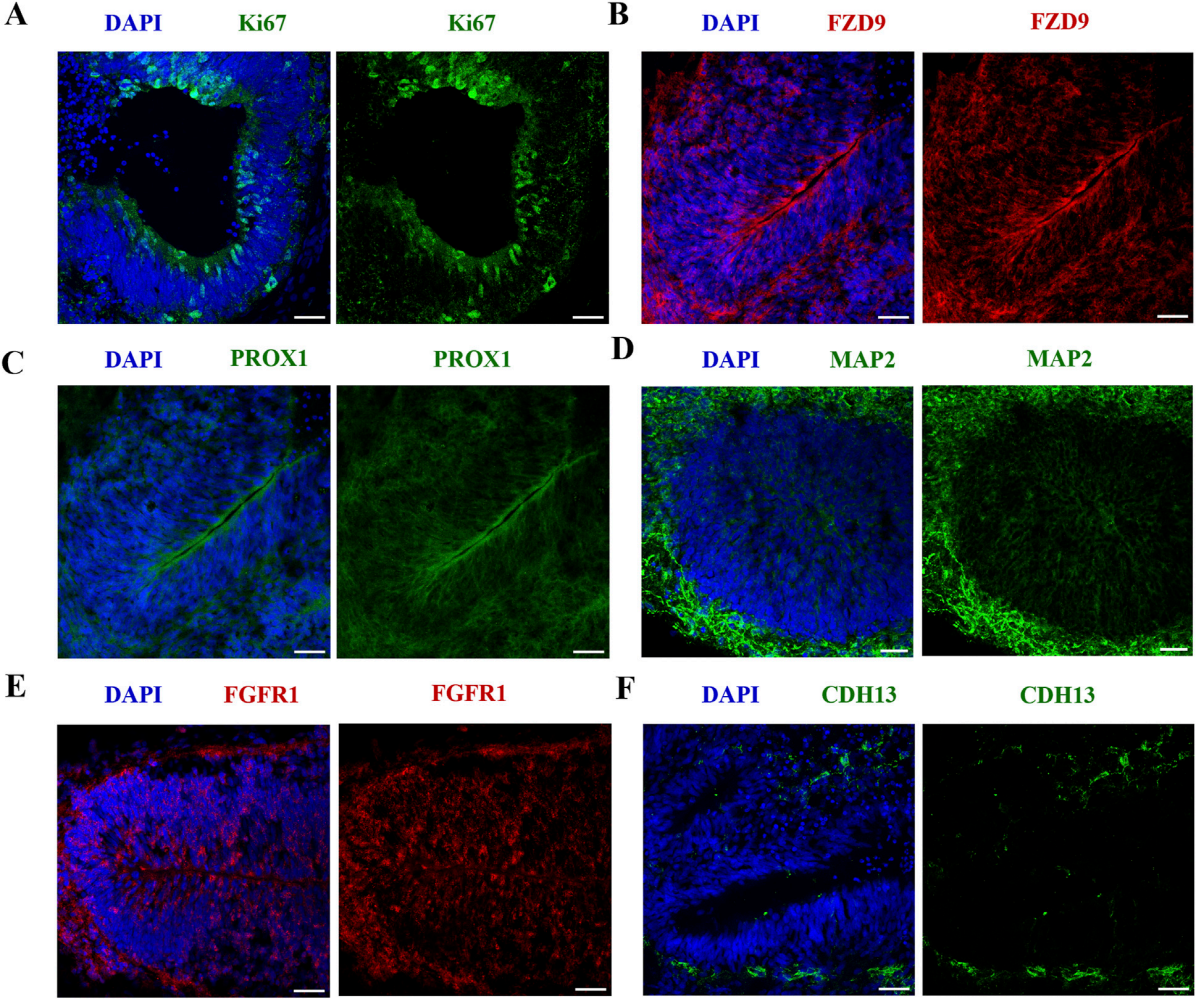
iPSC-derived cerebral organoids express markers specific to early brain regionalization and vital to development. Scale bar = 50 μm. **(A–F)** Immunofluorescent images were captured using confocal microscopy. **(A–C)** Staining of VEH organoids was performed for the expression of proliferation marker Ki67 **(A)** and regional markers FZD9 **(B)** and PROX1 **(C)** on D42. **(D–F)** VEH organoids also expressed neuronal marker MAP2 **(D)**, developmental markers FGFR1 **(E)** and CDH13 **(F)**.

Successful neural induction was confirmed by IF to characterize VEH organoids at D42 (Figure 2). VEH organoids stained positive for proliferation marker Ki67 (Figure 2A) and regional markers specific to hippocampal and cortical tissue (FZD9 and PROX1; Figures 2B, C). Neural induction was also confirmed by the presence of MAP2 (Figure 2D), which stains the neural cytoskeleton. Additionally, the organoids expressed markers vital to neurodevelopment (FGFR1 and CHD13; Figures 2E, F). These results exhibit that our organoids model aspects of neurogenesis, successful cortical differentiation, and developmental signatures of the fetal brain. Following initial characterization, the impact of nicotine exposure on these neurodevelopmental markers was also analyzed, however there were no significant differences (data not shown).

#### PNE acutely dysregulates aspects of neurogenesis and alters the expression of neural identity markers in the cortex

**FIGURE 3:**
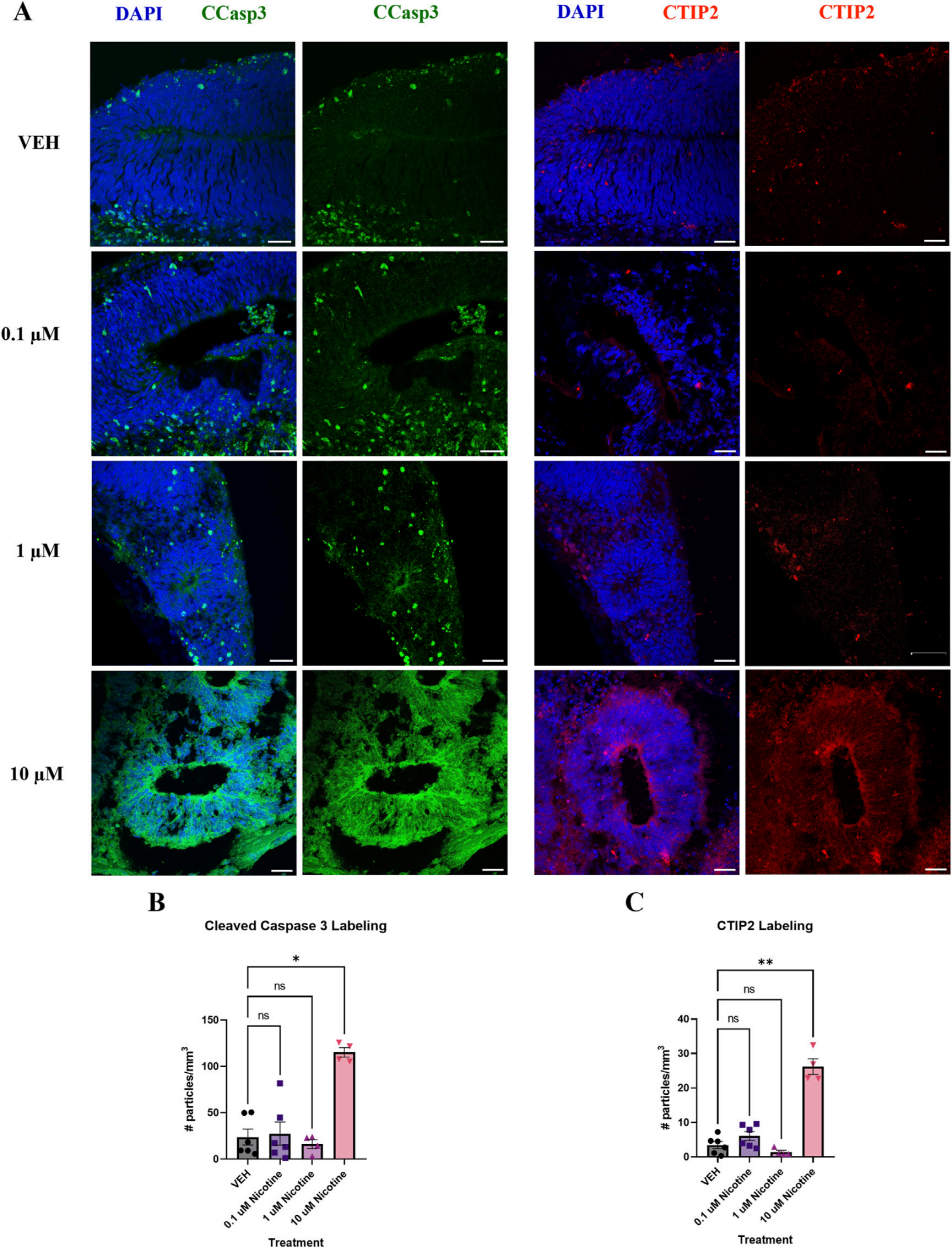
Nicotine significantly increases cell death and the number of early-born neurons on D42. **(A)** Immunofluorescent images captured using confocal microscopy of CCasp3 (green) and CTIP2 (red) in brain organoids treated with (0.1, 1 or 10 μM) nicotine or without (VEH) for 14 days. Scale bar = 50 μm. **(B, C)** Quantification of immunofluorescent images by the number of particles per area (mm^3^). **(B)** Organoids treated with 10 μM displayed a significant increase in apoptotic cell death, denoted by increased expression of CCasp3. **(C)** Nicotine significantly increased the presence of cortical layer marker CTIP2. Comparisons were made with Kruskal Wallis followed by Fisher’s LSD *post hoc* test. Data are mean ± SEM, n = 3 organoids per group; 20 total ROIs per marker, **p < 0.01, *p < 0.05, trending = *p* < 0.1, ns = not significant, *p* > 0.05. Each data point represents one ROI.

Following the 14-day nicotine treatment, there were changes in other markers used to characterize our model (Figure 3A). Indeed, there was a significant increase in cleaved caspase 3 (CCasp3) expression, a marker of apoptotic cell death (*H*_*(3,16*)_ = 9.174, *p* = 0.0271; Figure 3B). *Post hoc* analysis revealed an increase at 10 μM (*p* = 0.0114), with no effect observed at 0.1 or 1 μM (*p* > 0.05). We also examined deep-layer marker CTIP2, which was significantly increased in nicotine-treated organoids compared to VEH (*H*_*(3,16*)_ = 12.73, *p* = 0.0053; Figure 3C). The *post hoc* analysis demonstrated a similar result to CCasp3, with a significant increase at 10 μM (*p* = 0.0060) but not at lower doses of nicotine (*p* > 0.05). These findings demonstrate the toxicity of the 10 μM dose and the dose-dependent effect of nicotine on these specific markers. This suggests that these CCasp3 and CTIP2 may be more susceptible to the influences of nicotine at higher doses earlier in development, resulting in increased cell death and increased number of early-born neurons, whereas other developmental markers, such as those in Figure 2, are less sensitive to the effects of nicotine. Due to the observed toxicity at 10 μM, organoid viability was compromised, with limited experimental findings at D42 and no experimental analysis was performed at D180.

#### PNE has short-term effects on the expression of nAchRs implicated in anxiety and depression

**FIGURE 4:**
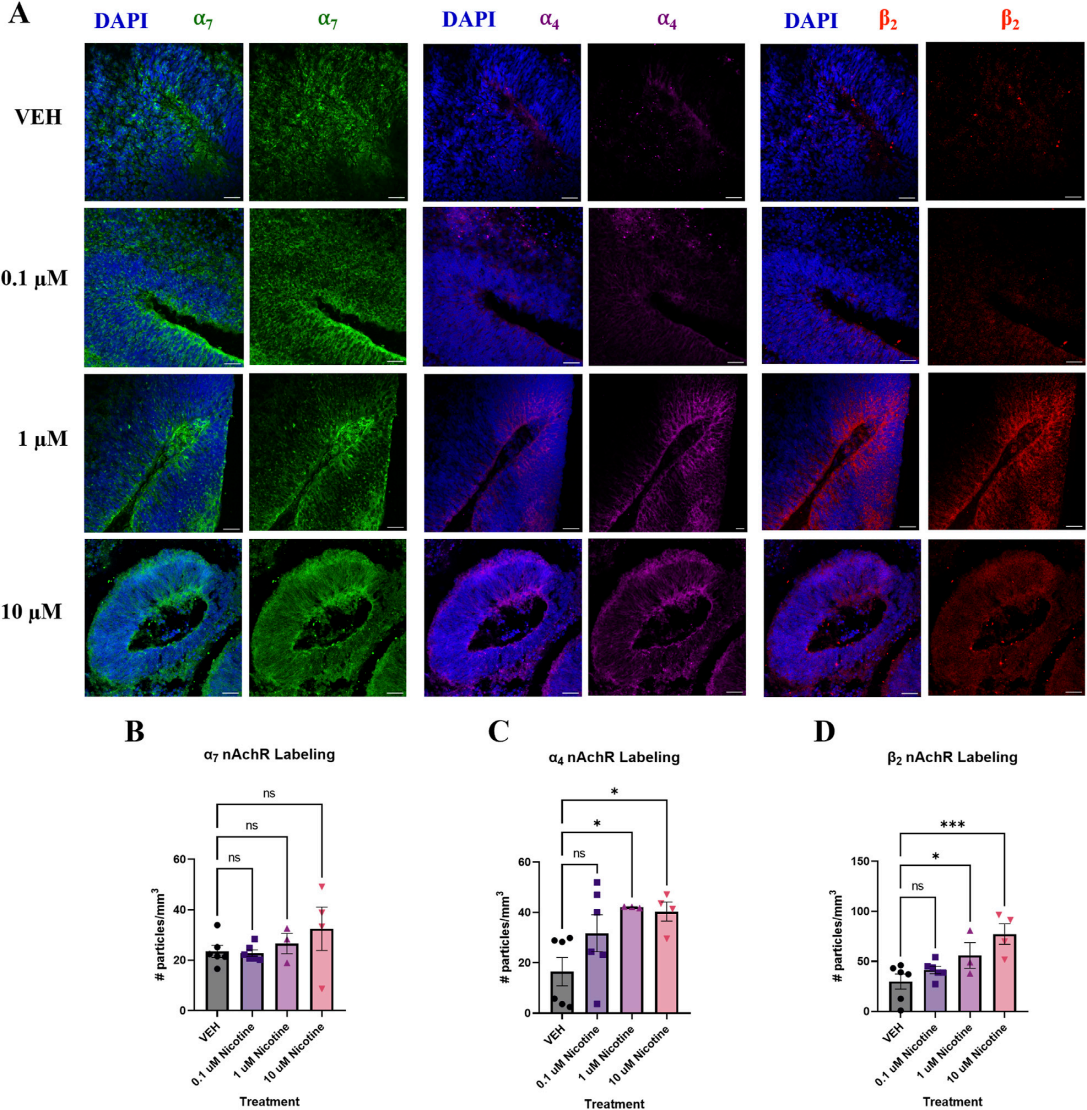
Nicotine selectively upregulates certain nAchR populations at D42. **(A)** Immunofluorescent images captured using confocal microscopy of α_7_ (green), α_4_ (magenta) and β_2_ nAchR (red) in brain organoids treated with (0.1, 1 or 10 μM) nicotine or without (VEH) for 14 days. Scale bar = 50 μm. (**B–D)** Quantification of immunofluorescent images by the number of particles per area (mm^3^). Nicotine did not affect α_7_ nAchR expression **(B)** but significantly increased α_4_ **(C)** and β_2_ **(D)** compared to VEH organoids. Comparisons were made with one-way ANOVA or Kruskal Wallis followed by Fisher’s LSD *post hoc* test. Data are mean ± SEM, n = 3 organoids per group; 18–20 total ROIs per marker, ***p < 0.001, *p < 0.05, ns = not significant, *p* > 0.05. Each data point represents one ROI.

Developmental nicotine exposure has been shown to dysregulate markers that are present in mood and anxiety disorders (Hudson et al., 2021; Jobson et al., 2019; Laviolette, 2021). To further investigate this, we employed IF to assess the impact of nicotine on these biomarkers at the protein level. To understand how nicotine may impact the expression of its target receptor, we looked at nAchR subunits that comprise the most abundant receptors in the cortex and are implicated in anxiety and depression; the α_7_, α_4_ and β_2_ nAchR subunits (Figure 4A). One-way ANOVA revealed that nicotine had no effect on α_7_ at any dose (F_(3,19)_ = 1.095, *p* = 0.3818; Figure 4B), but there was a significant increase in α_4_ (*H*_*(3,15*)_ = 7.424, *p* = 0.0449) and β_2_ (F_(3,15)_ *=* 6.706*, p* = 0.0043) nAchR subunits (Figures 4C, D) in nicotine treated organoids compared to VEH. Follow-up *post hoc* comparisons revealed a marked increase in α_4_ at the 1 (*p =* 0.0294) and 10 μM dose (*p* = 0.0205), but not at 0.1 (*p* > 0.05). Similarly, this was also seen in β_2_ at these doses (*p =* 0.0477; *p* = 0.0006; *p* > 0.05). Thus, nicotine upregulates the expression of some but not all nAchRs implicated in anxiety and depression at D42.

#### PNE acutely perturbs D1R and D2R expression

**FIGURE 5:**
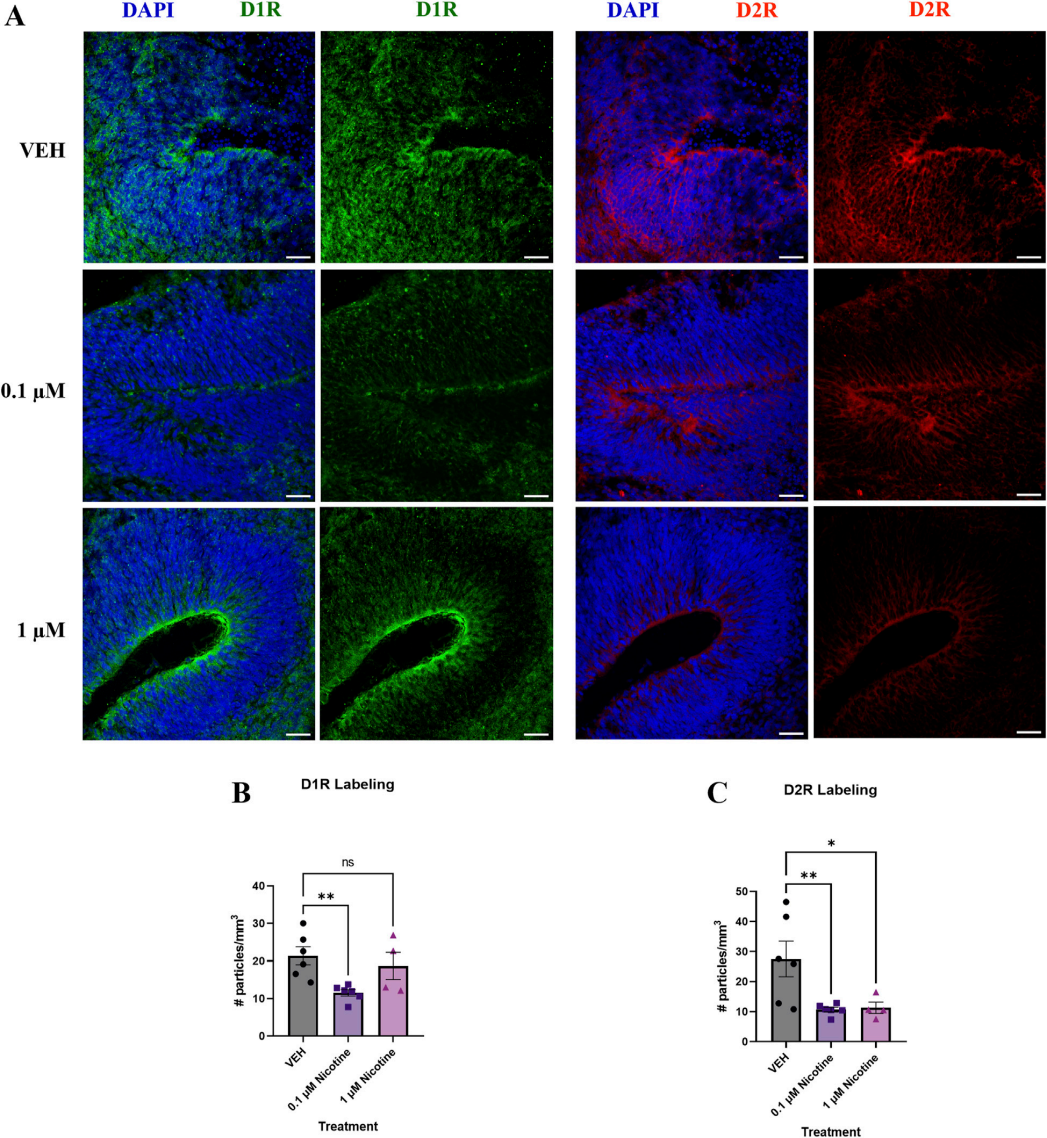
Nicotine induces significant alterations in dopaminergic receptors implicated in mood and anxiety disorders at D42. **(A)** Immunofluorescent images captured using confocal microscopy of D1R (green) and D2R (red) brain organoids treated with (0.1 or 1 μM) nicotine or without (VEH) for 14 days. Scale bar = 50 μm. **(B, C)** Quantification of immunofluorescent images by the number of particles per area (mm^3^). Compared to VEH, organoids treated with nicotine had significantly decreased levels of D1R at 0.1 μM **(B)** and D2R at 0.1 and 1 μM **(C)**. Comparisons were made with one-way ANOVA or Kruskal Wallis followed by Fisher’s LSD *post hoc* test. Data are mean ± SEM, n = 3 organoids per group; 16 total ROIs per marker, **p < 0.01, *p < 0.05, trending = *p* < 0.1, ns = not significant, *p* > 0.05. Each data point represents one ROI.

Since developmental nicotine exposure has been shown to disrupt dopaminergic signaling in brain regions associated with mood and anxiety control (Jobson et al., 2019), we decided to investigate changes in D1R and D2R expression following chronic nicotine exposure (Figure 5A). Following the 14 days of nicotine exposure, one-way ANOVA revealed a significant decrease in D1R receptor expression (F _(2,13)_ = 5.624, *p* = 0.0174; Figure 5B), with *post hoc* analysis suggesting this decrease occurred at 0.1 (*p =* 0.0061), with no effect at 1 μM (*p* > 0.05). Compared to VEH, one-way ANOVA indicated nicotine also significantly decreased D2R receptor expression (F_(2,13)_ = 6.023, *p* = 0.0141; Figure 5C). Further investigation using *post hoc* analysis exhibited that the decrease occurred at both 0.1 (*p* = 0.0077) and 1 μM (*p* = 0.0178). These results indicate that expression of these dopaminergic subtypes is influenced at lower doses of nicotine exposure, consistent with changes in protein expression seen in mood and anxiety disorders.

#### PNE disrupts GABAergic markers associated with cortical E/I balance at D42

**FIGURE 6:**
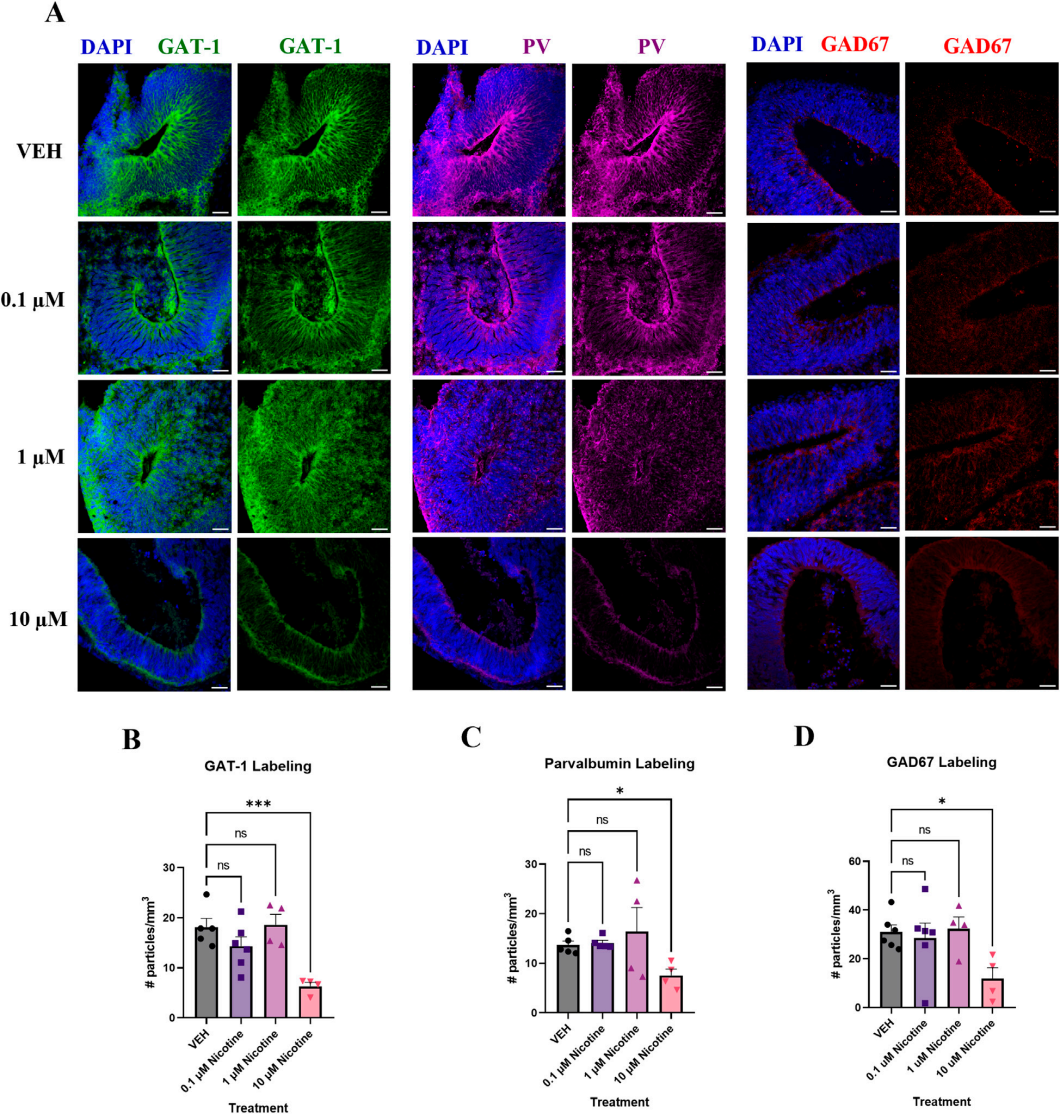
Nicotine induces significant deficits in markers vital to GABAergic synthesis, transport and signaling on D42. **(A)** Immunofluorescent images captured using confocal microscopy of GABA transporter GAT-1 (green), interneuron marker PV (magenta) and GABA synthesis marker GAD67 (red) in brain organoids treated with (0.1, 1 or 10 μM) nicotine or without (VEH) for 14 days. Scale bar = 50 μm. **(B–D)** Quantification of immunofluorescent images by the number of particles per area (mm^3^). At the 10 μM dose, organoids exhibited a significant decrease in GAT-1 **(B)**, PV **(C)**, and GAD67 **(D)** expression compared to VEH. Comparisons were made with one-way ANOVA or Kruskal Wallis followed by Fisher’s LSD *post hoc* test. Data are mean ± SEM, n = 3 organoids per group; 18–20 total ROIs per marker, ***p < 0.001, *p < 0.05, trending = *p* < 0.1, ns = not significant, *p* > 0.05. Each data point represents one ROI.

Finally, altered cortical E/I balance is a hallmark attribute of mood and anxiety disorders (Martin et al., 2020; Hashimoto, 2009) so we investigated the influence of nicotine on various GABAergic markers due to their role in cortical inhibition and neuron excitability (Figure 6A). A one-way ANOVA concluded that compared to VEH, nicotine significantly decreased the expression of GABA transporter GAT-1 (F_(3,15)_ = 8.778, *p* = 0.0013; Figure 6B), with *post hoc* analysis revealing a significant decrease at 10 (*p* = 0.0004), not 1 or 0.1 μM (*p* > 0.05). GABAergic alterations were also supported by a trend towards a decrease in PV interneurons in cortical regions of interest (*H*_*(3,14*)_ = 7.277, *p* = 0.0502; Figure 6C). Due to trending significance, a *post hoc* analysis was conducted and demonstrated a significant decrease in PV at 10 μM (*p* = 0.0350), but not lower doses of nicotine (*p* > 0.05). Finally, there was a trending decrease in levels of the GABA synthesis marker, GAD67 (*F*_*(3,16*)_ = 3.150, *p* = 0.0540; Figure 6D), which may suggest altered GABA neurotransmission in our organoids. Analogous to PV, *post hoc* comparisons were performed and revealed a significant deficit in GAD67 at 10 μM (*p* = 0.0157). These results suggest that at the protein level, higher doses of nicotine have a significant effect on GABAergic markers implicated in maintaining cortical E/I balance.

#### Nicotine-treated organoids endure significant transcriptomic changes in genes pertaining to nervous system development, neurogenesis and transcription regulation

**FIGURE 7:**
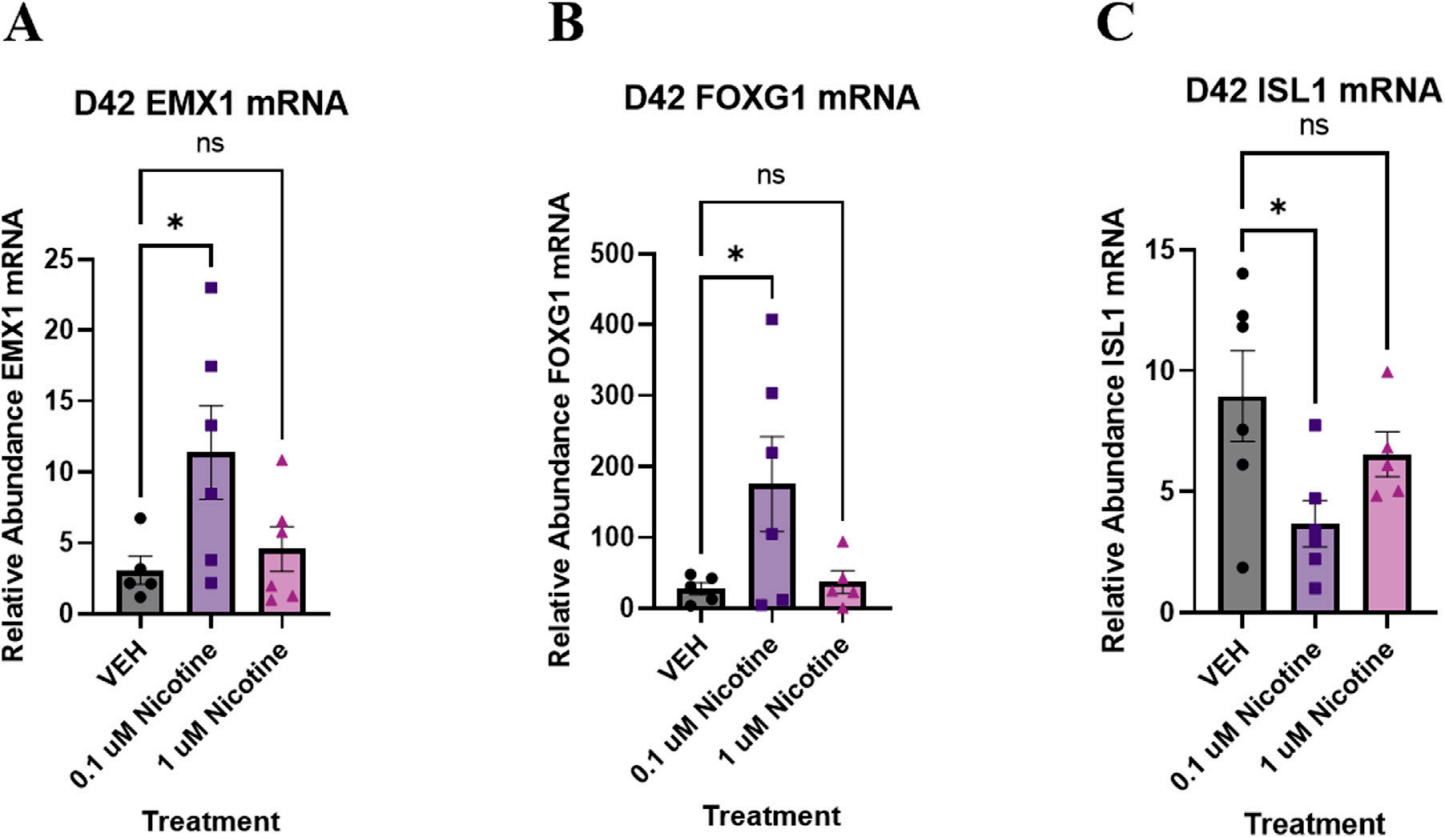
Nicotine elicits short-term changes in gene expression of various neural identity markers on D42. **(A–C)** Expression of relative abundance of mRNA of neural identity markers by qPCR in brain organoids exposed to nicotine (0.1, or 1 μM) or without (VEH) for 14 days. Relative mRNA abundance was calculated by normalizing the marker of interest to the geometric means of two housekeeping genes, *GAPDH* and *ACTB*. Organoids demonstrated a trending increase in cortical marker *EMX1* **(A)** and forebrain marker *FOXG1* **(B)**. There is a significant decrease in *ISL1*, a marker of embryonic development **(C)**. Comparisons were made with one-way ANOVA or Kruskal Wallis followed by Fisher’s LSD *post hoc* test. Data are mean ± SEM, n = 5–6 organoids per group, *p < 0.05, trending = *p* < 0.1, ns = not significant, *p* > 0.05.

Following IF analysis of proteins, nicotine-induced alterations were investigated at the transcriptomic level. Previously, chronic nicotine exposure has been associated with changes in markers pertaining to neural identity and forebrain development (Wang et al., 2018). Therefore, qPCR was used to evaluate changes in genes that may play a role in cortical development in conjunction with emotional and behavioral processes (Figure 7). One-way ANOVA suggested a trending increase in *EMX1* (F_(2,14)_ = 3.716, *p* = 0.0508; Figure 7A), which is implicated in the formation of the developing cerebral cortex. Due to trending significance, a *post hoc* analysis was performed and revealed a significant increase in *EMX1* at 0.1 (*p* = 0.0255) but not 1 μM (*p* > 0.05). Another gene implicated in the maturation of the cortex is *FOXG1*. One-way ANOVA also showed a trending increase in *FOXG1* in nicotine organoids compared to VEH (F_(2,13)_ = 3.582, *p* = 0.0577; Figure 7B). Due to trending significance, a *post hoc* analysis was completed and a significant increase in *FOXG1* was shown at 0.1 (*p* = 0.0350) but not 1 μM (*p* > 0.05). The final neural identify marker analyzed was *ISL1*, a gene vital to embryonic brain development. Analysis with one-way ANOVA demonstrated that nicotine organoids had a significant decrease in *ISL1* compared to VEH (F_(2,14)_ = 3.898, *p* = 0.0451; Figure 7C). Follow-up with *post hoc* comparisons determined there was a significant decrease at 0.1 (*p* = 0.0145) but not 1 μM (*p* > 0.05). These results demonstrate that genes underlying cortical development are more sensitive to lower doses of nicotine, which suggests that PNE may dysregulate cortical systems that are vital to emotional regulation.

**FIGURE 8:**
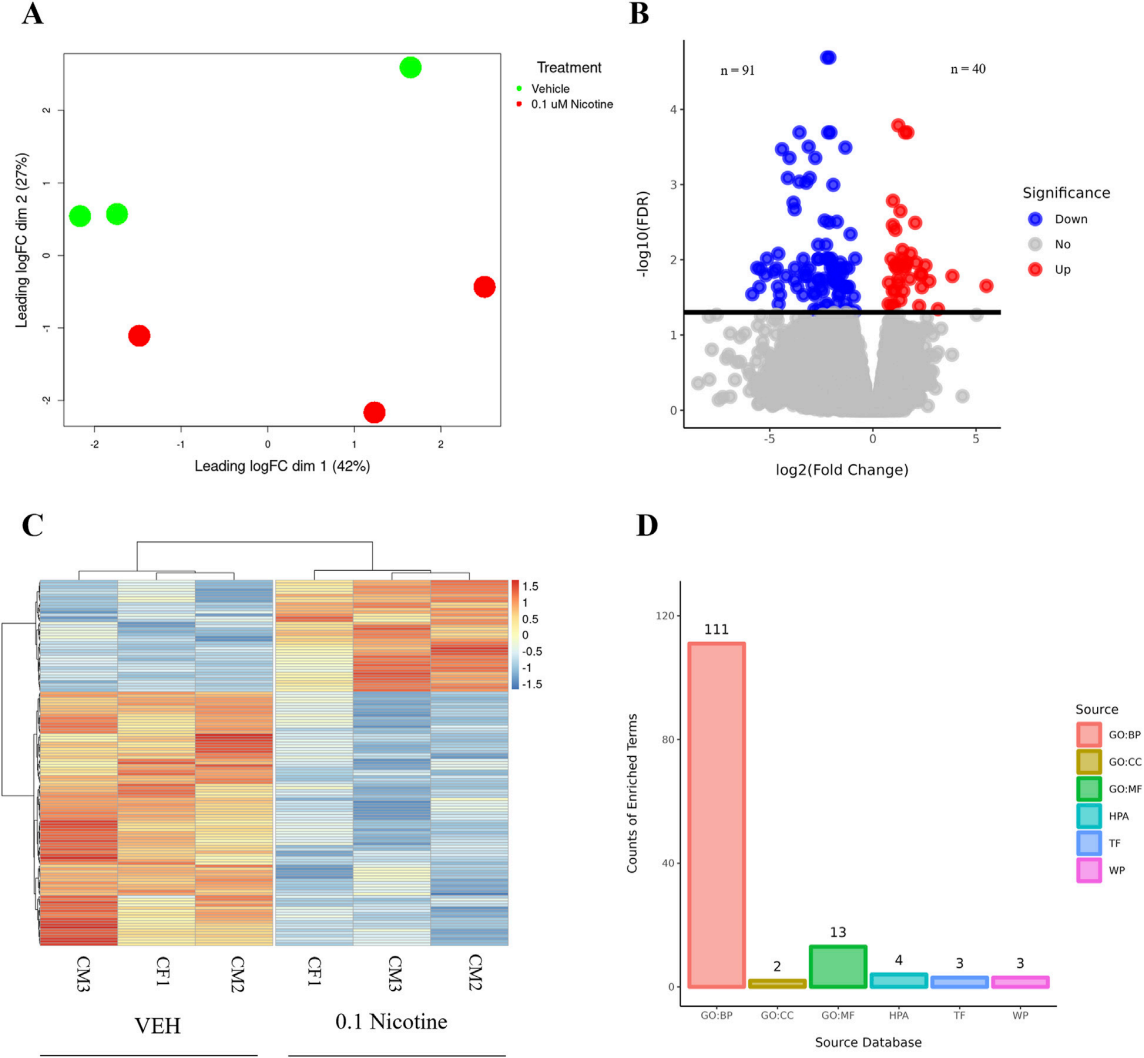
RNA-Seq differential gene expression and functional enrichment analysis of D42 VEH and 0.1 μM nicotine-treated organoids. **(A)** Multidimensional scaling plot demonstrating the separation of VEH (green) and 0.1 μM (red) nicotine-treated organoids. **(B)** Volcano plot illustrating the number of DEGs in VEH and 0.1 μM treated organoids. Upregulated genes in red (n = 40), downregulated genes in blue (n = 91). **(C)** Heatmap of log2 transformed normalized CPM values for all DEGs for VEH and 0.1 μM treated organoids. Values were centered, scaled across rows, and clustered using Ward’s clustering algorithm. Upregulated genes in red (n = 40), downregulated genes in blue (n = 91), n = 3 per group, FDR ≤ 0.05. **(D)** Overrepresented genes are categorized according to their functional characteristics quantified by the number of genes (counts) within a certain category. The categories originate from certain databases and include, from left to right, gene ontology (GO) biological processes (GO BP; 111), GO cellular component (GO CC; 2), GO molecular function (GO MF; 13), Human Protein Atlas (HPA; 4), Transfac (TF; 3) and Wikipathways (WP; 3).

**FIGURE 9:**
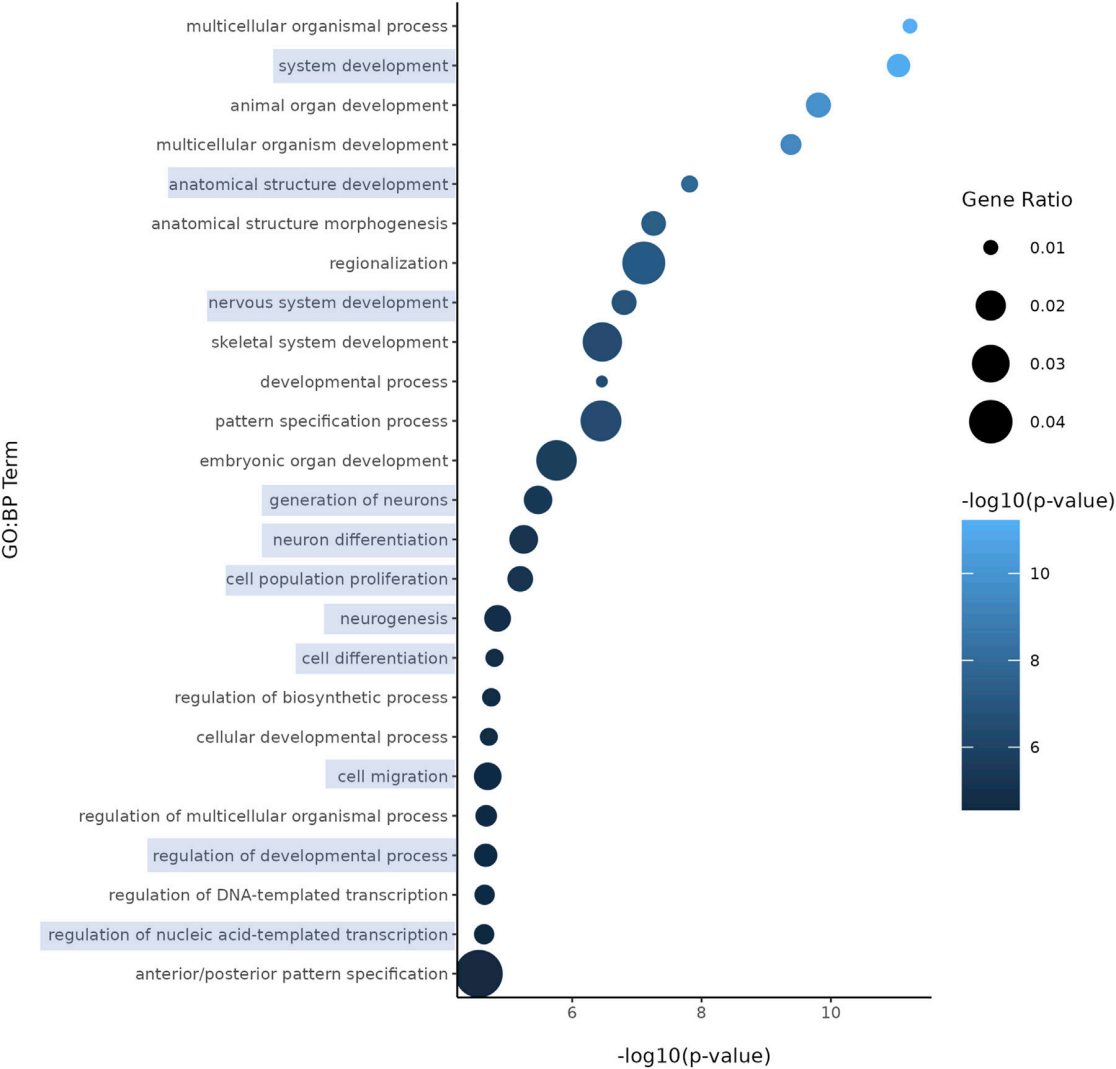
Enriched GO terms in D42 VEH and 0.1 μM nicotine-treated organoids. GO BP terms reveal that PNE elicits alterations in genes within processes such as nervous system development, neurogenesis, and other developmental processes. Circle size represents gene ratio and color represents fold change (-log10 transform of the adjusted *p*-value). Blue boxes represent terms of particular interest.

**FIGURE 10:**
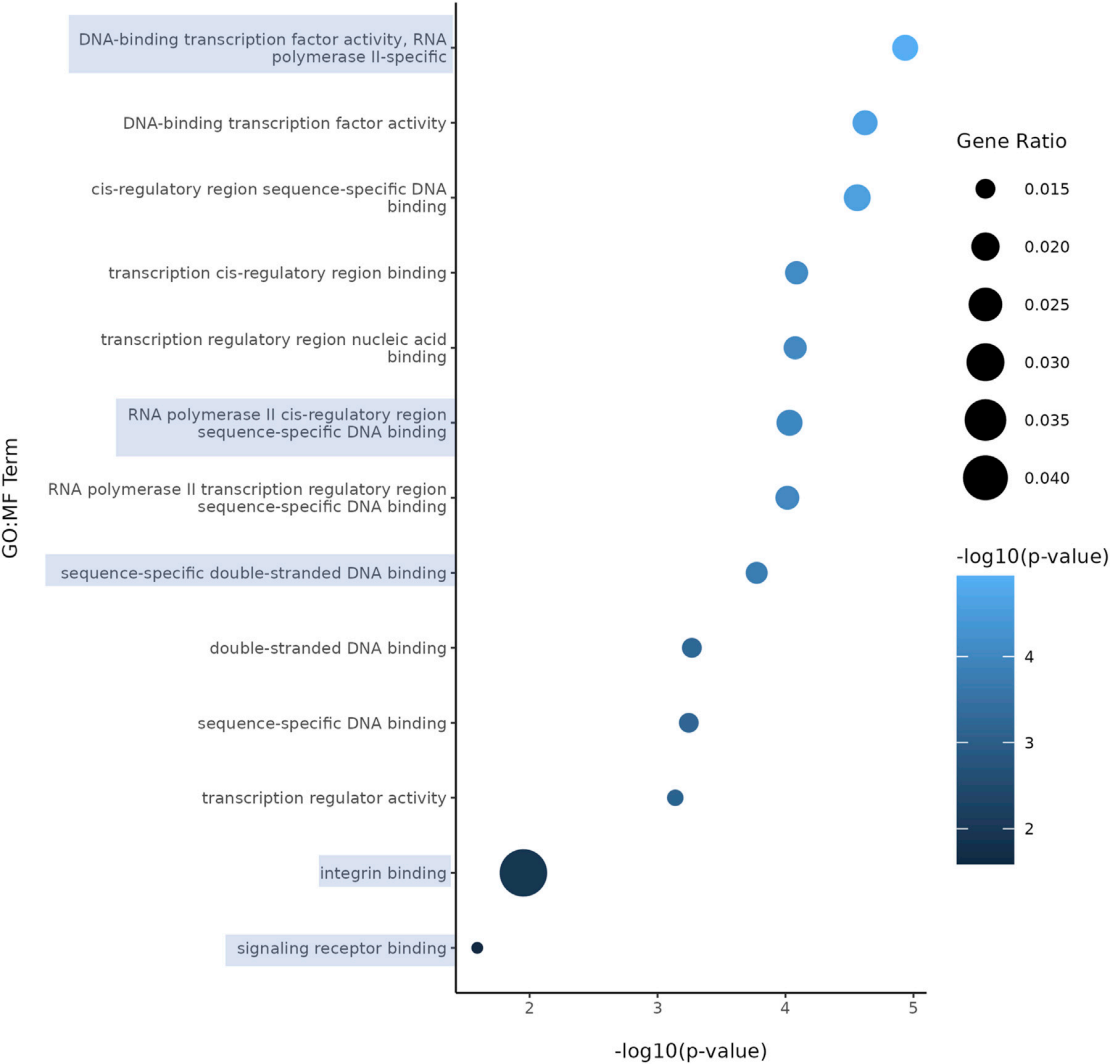
Enriched GO terms in D42 VEH and 0.1 μM nicotine-treated organoids. GO MF terms illustrate genes enriched in functions pertaining to DNA, signaling receptor and integrin binding activity. Circle size represents gene ratio and color represents fold change (-log10 transform of the adjusted *p*-value). Blue boxes represent terms of particular interest.

Both nicotine and mood/anxiety disorders have been known to cause cortical transcriptomic changes in clinical and preclinical studies (Lauterstein et al., 2016; Malki et al., 2015; Semick et al., 2020; Yoshino et al., 2021). Thus, it was of great interest to investigate if similar DEGs and transcriptomic alterations were captured using RNA-Seq in the brain organoids (Figure 8). The 0.1 µM dose was selected due to our D42 qPCR results as there were significant transcriptomic changes at 0.1 µM, but not 1 µM. To gain a sense of sample variation, a multidimensional scale plot was created (Figure 8A) and revealed that VEH and nicotine organoids separated more along the *x*-axis (42%) compared to the *y*-axis (27%). This indicates that the treatment groups were more different from each other than they were similar, with VEH grouping towards the top half of the multidimensional scale plot and nicotine organoids at the bottom. The short-term effects of nicotine exposure were examined by quantifying the number of DEGs and it was discovered that there were 91 downregulated genes and 40 upregulated genes when comparing nicotine-treated organoids to VEH (Figure 8B). Of these DEGs, when examining the top 20 (10 most downregulated and upregulated genes sorted by log fold change), upregulated genes include *NEUOROG2, EOMES* and the downregulated gene *CYP26C1* (Table 2). There were also multiple novel transcripts within the top 20 DEGs [denoted NA in Table 2]. Moreover, changes in DEG expression were summarized using a heat map (Figure 8C), which demonstrated that relative gene expression patterns between VEH and nicotine organoids looked quite different. There appears to be a larger number of genes that are transcribed more in the VEH (red) organoids whereas the nicotine organoids have a larger number of genes that are transcribed less (blue). Alternatively, there appears to be a smaller number of genes that are transcribed less in the VEH organoids whereas the nicotine organoids have a smaller number of genes that are transcribed more. To delve deeper into these classifications, GO analysis was conducted to examine specific terms that were enriched within BP and MF categories (Figure 8D). The GO database defines BP as specific physiological or cellular roles carried out by the gene whereas MF describes the molecular activity of a gene but does not provide any spatial information about where these functions occur in the cell. Analysis with GO BP, which had the highest number of counts, reveals terms enriched for several developmental processes (Figure 9). These terms involve organ (*p* = 1.56e-10), nervous system (*p* = 1.58e-7), and anatomical structural development (*p* = 1.53e-8). Likewise, a significant number of terms referring to neurogenesis (*p* = 1.42e-5) were present, including generation of neurons (*p* = 3.37e-6), neuronal differentiation (*p* = 5.62e-6), and cell migration (*p* = 2.02e-5). Several terms also included various regulatory processes like biosynthetic processes (*p* = 1.78e-5) and regulation of transcription (*p* = 2.30e-5). Like the transcription terms within BP, several terms were rereferring to transcription and DNA binding within the GO MF analysis, which further implicates the effect of nicotine on gene transcription (Figure 10). Integrin (*p* = 0.011), signaling receptor (*p* = 0.026), and transcription factor binding (*p* = 2.39e-5) were also principal terms reported in the MF analysis. Ultimately, the GO analysis elucidated that nicotine significantly impacted several BPs and MFs linked to embryonic development, transcription and gene expression.

**TABLE 2:** Top 20 DEGs in VEH and nicotine organoids.

Symbol	Gene	Log fold change	*p*-value	FDR	Significance
ENSG00000145626	*UGT3A1*	−5.85	1.40E-04	0.029	Down
ENSG00000250511	NA	−5.61	3.32E-05	0.013	Down
ENSG00000187553	*CYP26C1*	−5.50	1.01E-04	0.023	Down
ENSG00000279607	NA	−5.44	3.84E-05	0.013	Down
ENSG00000158022	*TRIM63*	−5.20	5.18E-05	0.016	Down
ENSG00000240990	*HOXA11-AS*	−5.13	1.65E-05	0.009	Down
ENSG00000248329	*APELA*	−4.79	4.44E-05	0.014	Down
ENSG00000174407	*MIR1-1HG*	−4.67	3.58E-05	0.013	Down
ENSG00000078399	*HOXA9*	−4.60	9.60E-05	0.023	Down
ENSG00000253293	*HOXA10*	−4.59	1.21E-05	0.008	Down
ENSG00000163508	*EOMES*	2.09	2.28E-05	0.011	Up
ENSG00000112333	*NR2E1*	2.24	2.29E-04	0.041	Up
ENSG00000178403	*NEUROG2*	2.31	4.84E-05	0.015	Up
ENSG00000087510	*TFAP2C*	2.35	5.21E-05	0.016	Up
ENSG00000251621	NA	2.37	1.04E-04	0.023	Up
ENSG00000286232	NA	2.55	2.79E-05	0.012	Up
ENSG00000168453	*HR*	2.73	7.45E-05	0.019	Up
ENSG00000280222	*NA*	3.16	2.55E-04	0.045	Up
ENSG00000280409	*LINC01101*	3.85	5.65E-05	0.016	Up
ENSG00000119614	*VSX2*	5.51	9.31E-05	0.022	Up

Abbreviations: FDR, false discovery rate; NA, novel transcript.

To follow up the GO analysis, we used VarElect to investigate the genetic overlap and phenotype-gene associations of our DEGs between three phenotypes of interest: nicotine exposure, anxiety, and depression (Stelzer et al., 2016; Table 3). All DEGs were imported into VarElect and ranked according to the elected phenotype. A list of the top 20 genes for each phenotype was generated. Within the list for each phenotype, five genes were consistent: dopamine transporter (*SLC6A3*), secreted phosphoprotein 1 (*SPP1*), nerve growth factor receptor (*NGFR*), histone deacetylase 9 (*HDAC9*) and insulin-like growth factor 2 (*IGF2*). These results imply that there is a shared genetic overlap between these phenotypes of interest. This may provide more insight as to how PNE and genetic variation within these genes underlie the incidence of mood and anxiety disorders.

**TABLE 3:** Common top 20 ranked genes related to nicotine, anxiety and depression phenotypes.

Gene symbol	Description	Rank (1–20) “Nicotine”	Rank (1–20) “Anxiety”	Rank (1–20) “Depression”
*SLC6A3*	Dopamine transporter	1	1	1
*SPP1*	Secreted phosphoprotein 1	2	8	17
*NGFR*	Nerve growth factor receptor	3	13	8
*HDAC9*	Histone deacetylase 9	6	11	18
*IGF2*	Insulin-like growth factor 2	9	7	11

### Long-term effects of PNE

#### PNE chronically perturbs proteins related to neuronal differentiation, dopaminergic and glutamatergic receptor expression

**FIGURE 11:**
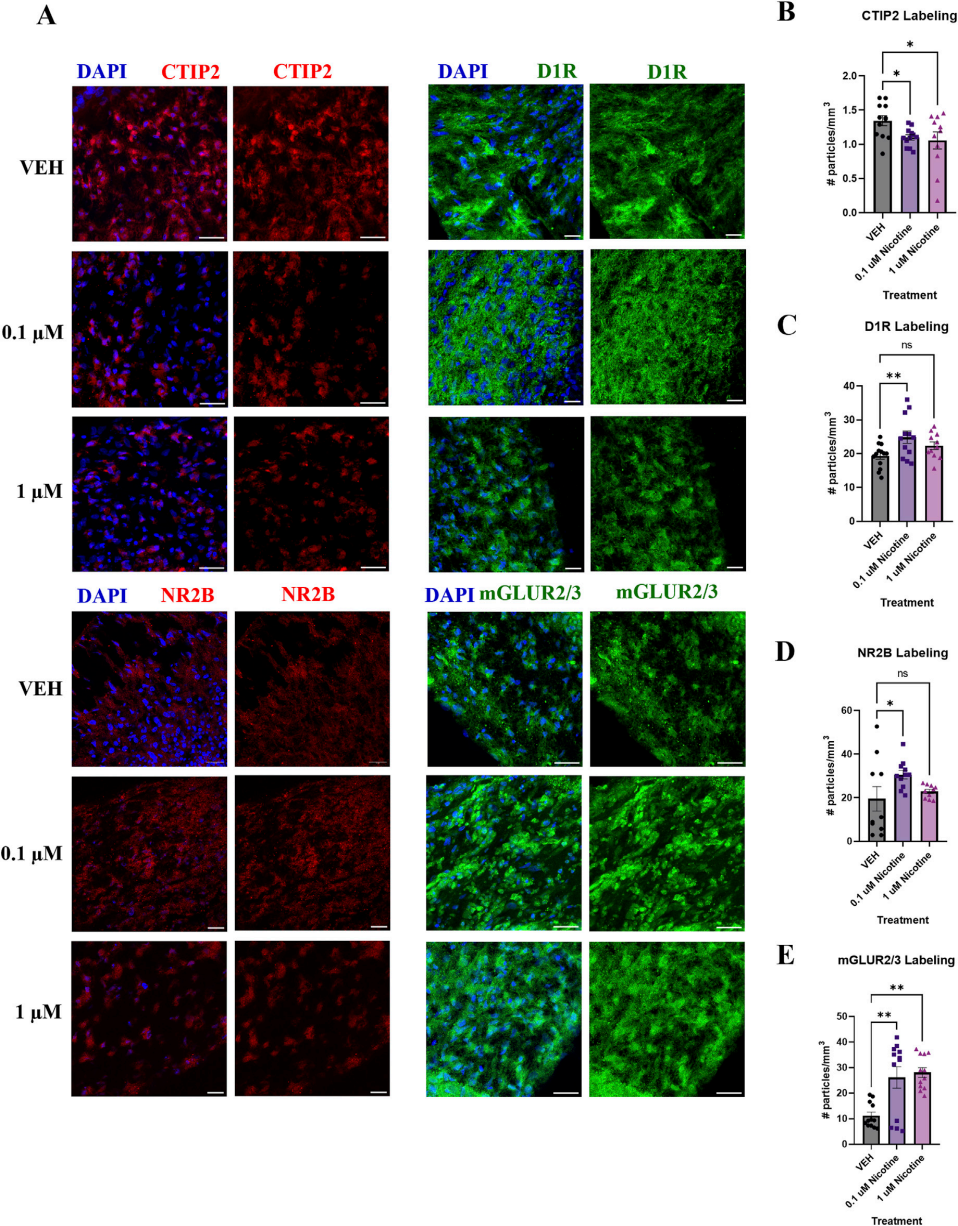
Nicotine induces long-term alterations in neuronal differentiation, dopaminergic and glutamatergic markers at D180. **(A)** Immunofluorescent images captured using confocal microscopy of CTIP2 (red), D1R (green), NR2B (red) and mGLUR2/3 (green) in brain organoids treated with (0.1 or 1 μM) nicotine or without (VEH) for 14 days. Scale bar = 50 μm. **(B–E)** Quantification of immunofluorescent images by the number of particles per area (mm^3^). Compared to VEH, organoids treated with nicotine had significantly decreased cortical layer marker CTIP2 levels at 0.1 and 1.0 μM **(B)**. Nicotine also significantly increased D1R **(C)** and glutamatergic markers NR2B **(D)** and mGLUR2/3 **(E)**. Comparisons were made with one-way ANOVA or Kruskal Wallis followed by Fisher’s LSD *post hoc* test. Data are mean ± SEM, n = 3 organoids per group; 31–36 total ROIs per marker, **p < 0.01, *p < 0.05, trending = *p* < 0.1, ns = not significant, *p* > 0.05. Each data point represents one ROI.

PNE has been associated with the emergence of mood and anxiety disorders later in the lives of children exposed to nicotine during pregnancy (Corrêa et al., 2022; Moylan et al., 2015). Therefore, we wanted to examine not only the immediate effects of nicotine but long-term outcomes during the later stages of organoid maturation (D180; Figure 11A). Unlike the significant increase reported at D42, at D180 we report a significant decrease in CTIP2 expression in nicotine-exposed organoids (F_(2,32)_ = 3.476, *p* = 0.0431; Figure 11B). *Post hoc* analysis stated a reduction in CTIP2 at both 0.1 (*p* = 0.0444) and 1 μM (*p* = 0.0213) in comparison to VEH. Due to the short-term effects of nicotine at D42, we also examined long-term changes in the dopaminergic receptor, D1R. One-way ANOVA also demonstrated a significant increase in D1R expression in nicotine organoids at D180 (F_(2,33)_ = 4.349, *p* = 0.0211; Figure 11C). *Post hoc* analysis identified an increase at 0.1 (*p* = 0.0059), but not 1 μM (*p* > 0.05). In addition to alterations in dopaminergic receptors, further long-term dysregulation was reported in glutamatergic receptors NR2B and mGLUR2/3. At D180, Kruskal–Wallis testing revealed a significant upregulation in NR2B (*H*_*(*2,28)_ = 6.136, *p* = 0.0465; Figure 11D). A *post hoc* was performed and showed an increase, specifically at 0.1 (*p* = 0.0333), but not 1 μM (*p* > 0.05). Finally, levels of mGLUR2/3 were significantly increased (*H*_(2,33)_ = 11.80, *p* = 0.0027; Figure 11E), with *post hoc* comparisons showing that mGLUR2/3 was elevated at both 0.1 (*p* = 0.0079) and 1 μM *(p* = 0.0013) compared to VEH. Altogether, these results signify long-lasting changes in neuronal differentiation, dopaminergic and glutamatergic proteins that persist until later stages of development following chronic nicotine exposure.

#### PNE has long-term transcriptomic effects on neural and cortical development

**FIGURE 12:**
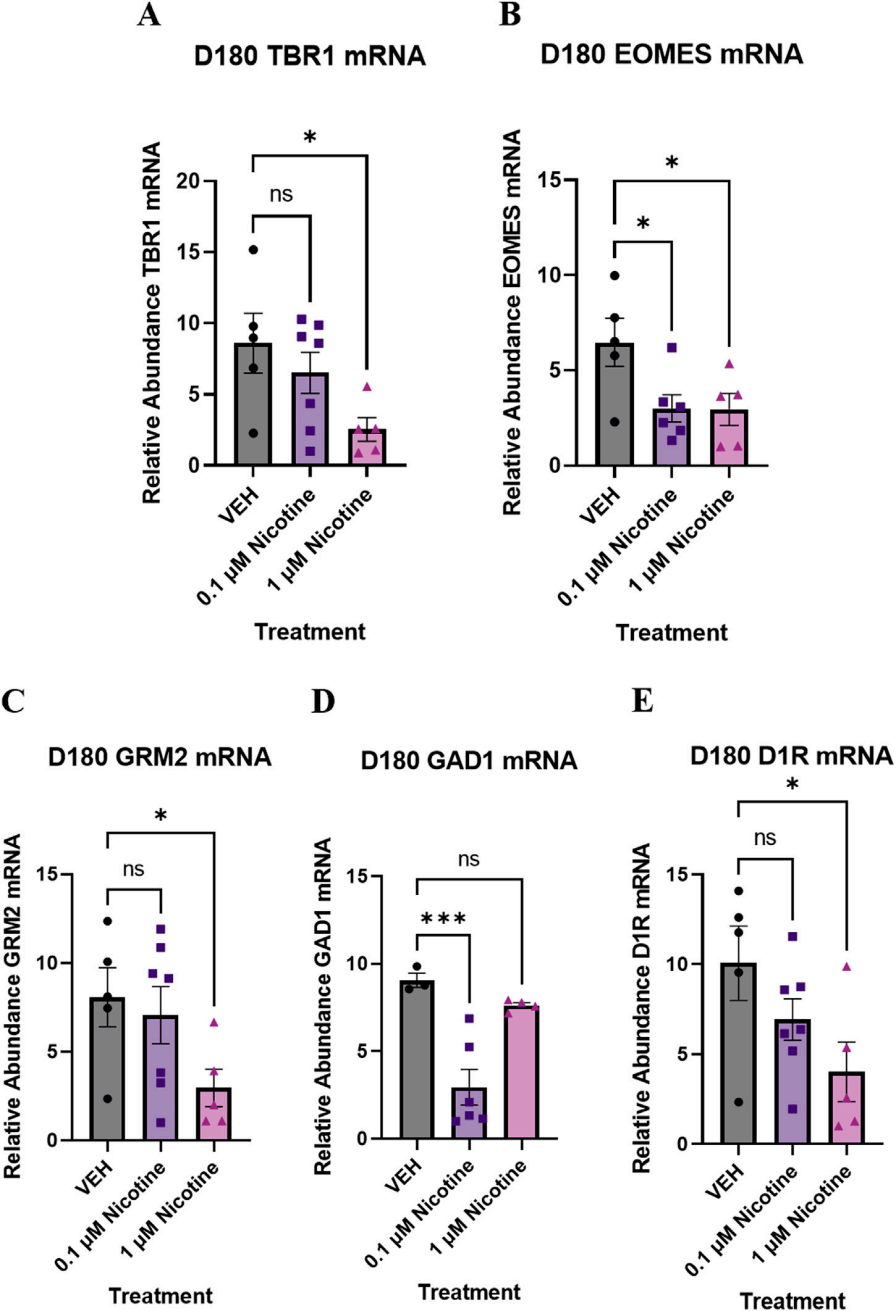
Nicotine elicits persistent, long-term changes in neural identity markers, glutamatergic, GABAergic and dopaminergic markers implicated in mood and anxiety disorders at D180. **(A–E)** Expression of relative abundance of mRNA by qPCR in mature D180 brain organoids exposed to nicotine (0.1, or 1 μM) or without (VEH) for 14 days. Relative mRNA abundance was calculated by normalizing the marker of interest to the geometric means of two housekeeping genes, *GAPDH* and *ACTB*. Nicotine was associated with a trending decrease in pre-plate marker *TBR1* **(A)** and a significant decrease in progenitor marker *EOMES* **(B)**. **(C)** Nicotine induced a trending, dose-dependent decrease in glutamatergic receptor *GRM2*. **(D)** Nicotine significantly downregulated the expression of GABA synthesis marker *GAD1*. **(E)** There was a significant decrease in *D1R* expression following nicotine exposure. Comparisons were made with one-way ANOVA or Kruskal Wallis followed by Fisher’s LSD *post hoc* test. Data are mean ± SEM, n = 3–7 organoids per group, ***p < 0.001, *p < 0.05, trending = *p* < 0.1, ns = not significant, *p* > 0.05.

To complement our IF results, the last set of experiments analyzed long-term changes in gene expression induced by nicotine exposure. We report that at D180, there was dysregulation in more than one neural identity marker. One-way ANOVA revealed a trend toward significantly decreased *TBR1*, a cortical pre-plate marker (F_(2,14)_ = 3.535, *p* = 0.0572; Figure 12A). Due to trending significance, a *post hoc* analysis was completed and revealed a significant decrease in *TBR1* at 1 (*p* = 0.0207) but not 0.1 μM (*p* > 0.05). Compared to VEH, there was also a significant decrease in neural progenitor marker *EOMES* (also known as *TBR2*; F_(2,13)_ = 4.441, *p* = 0.0339; Figure 12B). *Post hoc* analysis was completed and revealed a significant decrease in *EOMES* at 0.1 (*p* = 0.0206) and 1 μM nicotine (*p* = 0.0236). These results suggest that PNE has an enduring impact on neural and cortical development.

#### PNE elicits chronic alterations in glutamatergic, gabaergic and dopaminergic markers implicated in mood and anxiety disorders

Lastly, we quantified long-term changes in gene expression in glutamatergic, GABAergic, and dopaminergic markers implicated in mood and anxiety disorders. There was evidence of altered GABAergic and glutamatergic gene expression at D180, with a trending decrease in *GRM2*, the gene for mGLUR2 (F_(2,14)_ = 2.796, *p* = 0.0951; Figure 12C) and a significant decrease in *GAD1*, the gene for GAD67 (F_(2,10)_ = 14.55, *p* = 0.0011; Figure 12D). Due to trending significance in *GRM2*, a *post hoc* analysis was completed and revealed a significant decrease at 1 (*p* = 0.0447) but not 0.1 μM (*p* > 0.05). *Post hoc* comparisons were also performed for *GAD1* and showed a significant decrease at 0.1 μM (*p* = 0.0007) compared to VEH. There was no significant effect at 1 μM (*p* > 0.05). Consistent with dopaminergic perturbations at D42, one-way ANOVA described a trending decrease in *D1R* at D180 compared to VEH (F_(2,14)_ = 3.256, *p* = 0.0690; Figure 12E). Since trending significance was reported, *post hoc* comparisons were done and indicated a significant decrease at 1 (*p* = 0.0231) but not 0.1 μM (*p* > 0.05). These transcriptomic results signify that PNE unremittingly modifies neurotransmitter systems into later stages of neurodevelopment.

## Discussion

The association between developmental nicotine exposure and the emergence of mood and anxiety behaviors has been reported in various clinical and preclinical studies (Corrêa et al., 2022; Hudson et al., 2021; Jobson et al., 2019; Moylan et al., 2013; Moylan et al., 2015). However, the use of cerebral organoids to explicitly model PNE, offers a unique human-derived platform to build on prior research findings and bypass existing experimental limitations. Moreover, the relationship between PNE and the development of specific mood and anxiety molecular endophenotypes remains elusive and to our knowledge, has yet to be explored in cerebral organoids.

Consistent with previous research, chronic exposure to physiologically relevant doses of nicotine had widespread neuronal, molecular, and transcriptomic effects on our organoids (Notaras et al., 2021; Wang et al., 2018). We report that chronic nicotine exposure (0.1–10 μM) triggered apoptotic cell death and impacted the normal expression of various neural identity markers at D42 and D180. In terms of nicotine’s effect on its target receptors, there was short-term upregulation of α_4_ and β_2_ nAchR subunits. However, nicotine had no significant effect on α_7_ expression. Nicotine also dysregulated the expression of dopaminergic receptors, with long-lasting alterations in D1R expression persisting until D180. With regards to other neurotransmitter systems, nicotine affected glutamatergic and GABAergic markers of interest, which implies shifted E/I balance in the cortex and remained until the later stages of organoid maturation. Finally, RNA-Seq revealed substantial transcriptomic changes in organoids treated with 0.1 μM nicotine. Numerous BPs and MFs were differentially expressed, specifically in targets involving nervous system development, neurogenesis, and transcription activity.

Nicotine exposure has been shown to disrupt many facets of fetal neurogenesis, including changes in neuronal differentiation and apoptosis (Aoyama et al., 2016; Dwyer et al., 2009; Smith et al., 2010; Wang et al., 2018). For instance, chronic PNE has been shown to disrupt the cell cycle of neural progenitors and accelerate neuronal differentiation (Aoyama et al., 2016; Takarada et al., 2012). To further support this claim, compared to controls, 1 μM and 10 μM nicotine-treated organoids on a chip demonstrated increased short-term expression of CTIP2, an early-born neuronal marker, and CCasp3, a member of the caspase family implicated in neuronal death in neural syndromes (Wang et al., 2018). These alterations were accompanied by no change in the number of neural progenitor cells, which suggests nicotine disrupted cortical neuronal layering by the induction of premature differentiation. Wang’s dose-dependent descriptions of increased differentiation and apoptosis at early developmental stages align with our D42 IF findings. Similarly, at D42, we report increased CCasp3 and CTIP2 at 10 μM nicotine, which also indicates short-term, dose-dependent increases in apoptosis and neuronal differentiation at higher nicotine concentrations. These findings suggest nicotine exposure has a dual effect on the developing brain, simultaneous apoptosis, and compensatory neuronal differentiation. Unlike Wang’s study, which focused on early developmental stages of nicotine exposure, we also report long-term neurodevelopmental changes at D180, showing a significant reduction of CTIP2 at 1 μM, indicating a different dose response relationship over time. Although there is an initial compensatory response at D42, these findings suggest this mechanism may not be sufficient to sustain long-term neuronal health, leading to different outcomes over prolonged exposure. The differential response may indicate a biphasic effect, where high doses initially stimulate differentiation, but long-term exposure to lower doses disrupts it. Our findings provide insight to both dose-dependent and time-dependent effects of nicotine on neurodevelopment, emphasizing the need to carefully consider exposure levels during gestation. Understanding the impact of nicotine exposure at various stages of development can provide further insight to its lasting effects, potentially influencing mood and anxiety disorders. Apart from alterations in neurogenesis, PNE has also been found to impact the expression of various categories of neural identity markers. For example, the same study by Wang and colleagues (2018) reported differential expression of preplate marker *TBR1*, forebrain marker *FOXG1* and increased expression of hindbrain marker *ISL1* in their brain organoids (Wang et al., 2018). Indeed, mice with deficiencies in certain neural identity markers, like cortical marker *EMX1*, demonstrate lower levels of depressive behaviors, denoted by reduced immobility time in the forced swim test and reduced anxiety in the light/dark box and elevated plus maze (Cao and Li, 2002). Additionally, mice exposed to PNE demonstrate decreased PFC expression of *EOMES*, or *TBR2*, as well as cognitive and emotional deficits in adulthood (Aoyama et al., 2016). Our qPCR data confirms a significant increase in *EMX1* and *FOXG1* alongside a significant decrease in *ISL1* at D42. This suggests dysregulated development of neuronal populations comprising the forebrain and hindbrain. Additionally, we report significant reductions in *TBR1* and *EOMES* at D180 signifying long-term changes in cortical development. Overall, our qPCR results suggest nicotine-induced dysregulation of neurogenesis and numerous cortical markers that persist until D180. Thus, further studies examining these alterations underlying cortical development may help to understand the impact of PNE on the behavioral dysfunctions of the offspring when these developmental pathways mature.

α_4_β_2_ and α_7_ are the most abundant nAchRs in the cortex and are implicated in various cognitive and attentional functions (Alkam and Nabeshima, 2019; Livingstone et al., 2010). Results from preclinical studies have demonstrated that chronic PNE from gestational day 7–21 elevates α_4_, α_7_ and β_2_ mRNA expression in the rat cortex and hippocampus (Shacka and Robinson, 1998). This has also been seen in α_4_ and α_7_ mRNA of human fetuses exposed to nicotine during pregnancy (Falk et al., 2005). Our IF results revealed significantly increased α_4_ and β_2_ nAchR protein expression following nicotine exposure at D42, which can have functional implications in the development of mood and anxiety disorders (Saricicek et al., 2012). For instance, abnormalities in α_4_β_2_ nAchR expression and function, specifically in the PFC and hippocampus, may contribute to these disorders due to their ability to modulate GABA release (Fogaça and Duman, 2019; Freund et al., 1988; Kutlu and Gould, 2015; Lu et al., 1998). Therefore, altered nAchR levels could lead to an imbalance in GABAergic neurotransmission, alter mood and anxiety-related brain circuitry and lay the foundation for altered E/I levels previously reported in the literature (Fogaça and Duman, 2019; Sequeira et al., 2009). Interestingly, there was no change in α_7_ expression which was unexpected due to its role in regulating cortical glutamate release (Livingstone et al., 2010). This insignificant effect in α_7_ was especially surprising given our reports of immediate and long-term perturbations in GABA receptor expression in our IF and qPCR analyses. However, α_7_ nAchR subunits desensitize more rapidly than α_4_ and β_2_ and have a lower affinity for nicotine (Dwyer et al., 2019; Fenster et al., 1999). Additionally, to our knowledge, the specific timing that these subtypes of nAchRs appear in development has yet to be identified in cerebral organoids, which further complicates temporal analyses in this PNE model. Nevertheless, the present findings may help elucidate the correlation between the temporal specificity of nicotine exposure on the circuitry of the developing fetal brain and the future emergence of mood and anxiety behaviors.

In comparison to other neurotransmitter systems, the effect of PNE on dopaminergic receptors in the fetal brain is unclear. However, as reported in a preclinical rodent model of adolescent nicotine exposure, another critical period of neurodevelopment, rodents chronically exposed to nicotine demonstrated significantly less D1R expression levels in the PFC compared to VEH (Jobson et al., 2019; Laviolette, 2021). This was concurrent with no significant changes in D2R expression. Likewise, other animal studies have demonstrated dopaminergic hypofunction in the neocortex resulting from PNE as well as decreased dopaminergic metabolites (Ernst et al., 2001; Muneoka et al., 1997; Muneoka et al., 1999). Dopamine is suggested to be involved in anxiety-like behaviors and is involved in the regulation of emotion, therefore, this hypofrontality is also linked to the pathology of anxiety and depression (Muneoka et al., 1999; Zarrindast and Khakpai, 2015). Our D42 IF findings are consistent with previous reports of decreased D1R expression following nicotine exposure. Unlike previous preclinical studies that have failed to detect differences in D2R following nicotine exposure, we also report significant decreases in D2R at D42. Our qPCR data also revealed that reductions in *D1R* persist until D180, while IF demonstrated an increase in D1R protein expression. This may suggest that D1R is more vulnerable long-term to the effects of nicotine in comparison to D2R and altered dopaminergic signaling persists past the point of initial exposure. Given that various neurotransmitter systems, including acetylcholine and dopamine, occupy trophic roles in the development of the central nervous system, it is important to characterize when these receptors are susceptible to manipulation in the organoid model and in turn, how they could evoke long-term neurodevelopmental consequences for the offspring (Wickström, 2007).

In tandem with dopaminergic alterations, other cortical biomarkers of MDD resulting from excess cholinergic signaling are hyperglutamatergia and decreased GABAergic signaling, which subsequently disrupts E/I balance (Dwyer et al., 2009; Fogaça and Duman, 2019; Hashimoto, 2009; Livingstone et al., 2010; Martin et al., 2020; Nobis et al., 2020). Increased glutamate levels have been reported in *postmortem* cortical tissue of individuals with MDD, suggesting aberrant glutamatergic transmission underlying features of MDD (Hashimoto, 2009). In recent years, animal studies have reported antidepressant effects resulting from ketamine, an N-methyl-D-aspartate antagonist, in reducing immobility time in the forced swim test and shock-induced behavioral changes (Chaturvedi et al., 1999; Hashimoto, 2009; Yilmaz et al., 2002). Notably, clinical studies have demonstrated the antidepressant effect of ketamine, in treatment resistant MDD, and are investigating other glutamatergic receptors (e.g., α-amino-3-hydroxy-5-methyl-4-isoxazolepropionic acid receptor, mGLURs) as additional therapeutic targets (Hashimoto, 2009; Zarate et al., 2006). In terms of reduced inhibitory synaptic transmission, marked reductions in GABA synthesis enzymes and decreased size and density of GABAergic interneurons have been found in the dorsolateral PFC of depressed individuals (Fogaça and Duman, 2019; Karolewicz et al., 2010). Likewise, a mouse model of PNE confirmed a shift towards excitation in the E/I balance, denoted by a dose-dependent decrease in cortical GABAergic neurons (Martin et al., 2020). Similar findings of reduced GABA and receptor functioning have been described in preclinical stress models (Sanacora et al., 1999). Our results are consistent with previously reported GABA and glutamate dysfunction in MDD. Our IF analysis exhibited GABAergic deficits, specifically decreased GAT-1, PV and GAD67 at D42 and increased expression of glutamatergic markers NR2B and mGLUR2/3 at D180. Our D180 qPCR results also revealed alterations in *GRM2* and *GAD1*, indicating that long-lasting changes are occurring at the level of gene transcription. Nonetheless, to strengthen the causal link between nicotine-related errors in E/I neurotransmission and phenotypes of mood and anxiety disorders, future studies are required to explore the electrophysiological impacts on neuronal activity states within nicotine-exposed cerebral organoids, to fully understand the impacts of these molecular alterations on neuronal activity states.

Finally, research has shown that PNE significantly impacts aspects of nervous system development such as the generation, proliferation, differentiation, and migration of neurons (Mizrak, 2019; Wang et al., 2018). This has been documented in RNA-Seq analysis of *postmortem* PFC tissue from fetuses of smoking mothers which revealed increased expression of genes involved in neurodevelopment (Semick et al., 2020; Sherafat et al., 2021). This exposure to nicotine underlies changes in various neurotrophic factors, such as brain-derived neurotrophic factor and NGF that are essential for the growth and survival of neurons (Lauterstein et al., 2016). In turn, these modifications have the capacity to influence the human genome and epigenome, which may increase the occurrence of MDD, and suggests a genetic overlap between nicotine exposure and mood disorders (Dome et al., 2010; Lauterstein et al., 2016). For example, there is evolving evidence that suggests abnormal transcriptional regulation is a crucial component of mood disorders (Hobara et al., 2010). Mainly, a theory surrounding the evolution of MDD is that chronic stress induces alterations in the transcriptional regulation of growth factors, which leads to impaired neurogenesis (Malki et al., 2015). Similar findings were reported in human *postmortem* brain tissue where significant DEGs were enriched in pathways relating to neurodevelopment such as NGF, neurotrophin, and integrin signaling (Yoshino et al., 2021). There were also significant genes in specific function and disease pathways such as psychological disorders and nervous system development (Yoshino et al., 2021). Furthermore, repeated nicotine exposure can exert various epigenetic modifications such as the activity of nicotine-responsive transcription factors and inhibition of histone deacetylases (HDACs), which greatly modify gene expression (Volkow, 2011). Changes in HDACs are also seen in MDD. For instance, compared to nonpsychiatric controls, Hobara et al., 2010 reported decreased expression of HDAC9 mRNA in patients with mood disorders. This further associates transcriptional alterations as a focal point within mood disorders. Our RNA-seq results are consistent with previous findings in transcriptional studies of PNE and MDD, with many of our GO BP terms representing nervous system development, neurogenesis, and regulation of developmental/transcriptional processes. As for GO MF, all the terms were related to transcription factor activity, DNA, integrin or signaling receptor binding. This provides a better understanding of how nicotine influences MFs that are also altered in mood and anxiety disorders. We also reported five overlapping DEGs shared between nicotine, anxiety and MDD phenotypes: *SLC6A3, SPP1, NGFR, HDAC9* and *IGF2*. These genes were altered following nicotine exposure but also have a role in neurodevelopment, mood and anxiety disorders or closely interact with genes related to these phenotypes (Fan et al., 2020; Hobara et al., 2010; Lauterstein et al., 2016; Luo et al., 2015; Rafikova et al., 2021). Comprehensively, our results strengthen the genetic association between neurodevelopmental and transcriptional abnormalities resulting from PNE and the basis of mood and anxiety molecular endophenotypes. Future efforts are required to fully validate and characterize novel DE transcripts, their underlying function in the cortical transcriptome and their role in neuropsychiatric disorders.

## Conclusion

Using cerebral organoids, the present study aimed to validate a novel application of a human-derived *in vitro* model, to better comprehend the emergence of neurodevelopmental abnormalities and the manifestation of neuropsychiatric molecular endophenotypes resulting from chronic PNE. The advent of iPSC technology coupled with molecular analyses provided a framework to examine long-lasting alterations in fetal neurodevelopment, modifications in receptors vital to mood and anxiety pathophysiology and changes to the cortical transcriptome. Understanding how environmental drug exposure during pregnancy alters early cortical development and the resulting changes in biomarkers may raise awareness to the dangers of electronic nicotine delivery systems and provide a basis for the etiology of mood and anxiety disorders in human-based models. In the future, this will provide a platform for patient-specific treatments and finding appropriate and efficacious interventions to improve the outcomes of the offspring, who without choice, struggle with these neuropsychiatric disorders long-term.

## Data Availability

The datasets generated for this study can be found in the National Library of Medicine, Accession: PRJNA1137246; ID: 1137246: https://www.ncbi.nlm.nih.gov/bioproject/PRJNA1137246/.

## Glossary

ANOVA: Analysis of variance
BP: Biological process
CCasp3: Cleaved caspase 3
CDH13: Cadherin-13
cDNA: Complementary DNA
CTIP-2: COUP-TF-interacting protein 2
Cq: Cycle quantification
DEGs: Differentially expressed genes
DEPC: Diethyl pyrocarbonate
D1R: Dopamine 1 receptor
D2R: Dopamine 2 receptor
D42: Day 42
D180: Day 180
EB: Embryoid body
E/I: Excitatory/inhibitory
EOMES: Eomesodermin
FDR: False discovery rate
FGFR1: Fibroblast growth factor receptor 1
FOXG1: Forkhead-box G1
FZD9: Frizzled-9
GABA: γ-aminobutyric acid
GAD1: Glutamate decarboxylase 1
GAD67: Glutamic Acid Decarboxylase 67
GAT-1: GABA transporter type 1
GO: Gene ontology
GRM2: Metabotropic glutamate receptor 2
HDAC: Histone deacetylase
IF: Immunofluorescence
iPSCs: Induced pluripotent stem cells
ISL1: ISL LIM homeobox 1
Ki67: Antigen Kiel 67
LSD: Least squared difference
MAP2: Microtubule-associated protein 2
MDD: Major depressive disorder
MF: Molecular function
mGLUR2/3: Metabotropic glutamate receptor 2/3
nAchRs: Nicotinic acetylcholine receptors
NGF: Nerve growth factor
NR2B: NMDA receptor subunit 2B
PBS-T: Phosphate buffered saline with Tween 20
PFC: Prefrontal cortex
PNE: Prenatal nicotine exposure
PROX1: Prospero homeobox 1 protein
PV: Parvalbumin
qPCR: Real-time quantitative polymerase chain reaction
RNA-Seq: Ribonucleic acid sequencing/RNA sequencing
ROI: Region of interest
VEH: Vehicle
